# A Bayesian method for comparing and combining binary classifiers in the absence of a gold standard

**DOI:** 10.1186/1471-2105-13-179

**Published:** 2012-07-27

**Authors:** Jonathan M Keith, Christian M Davey, Sarah E Boyd

**Affiliations:** 1 School of Mathematical Sciences, Monash University, Victoria 3800, Australia

**Keywords:** Binary classifier, Bayesian methods, Protein sub-cellular localisation, Diagnostic tests, Genome wide association studies.

## Abstract

**Background:**

Many problems in bioinformatics involve classification based on features such as sequence, structure or morphology. Given multiple classifiers, two crucial questions arise: how does their performance compare, and how can they best be combined to produce a better classifier? A classifier can be evaluated in terms of sensitivity and specificity using benchmark, or gold standard, data, that is, data for which the true classification is known. However, a gold standard is not always available. Here we demonstrate that a Bayesian model for comparing medical diagnostics without a gold standard can be successfully applied in the bioinformatics domain, to genomic scale data sets. We present a new implementation, which unlike previous implementations is applicable to any number of classifiers. We apply this model, for the first time, to the problem of finding the globally optimal logical combination of classifiers.

**Results:**

We compared three classifiers of protein subcellular localisation, and evaluated our estimates of sensitivity and specificity against estimates obtained using a gold standard. The method overestimated sensitivity and specificity with only a small discrepancy, and correctly ranked the classifiers. Diagnostic tests for swine flu were then compared on a small data set. Lastly, classifiers for a genome-wide association study of macular degeneration with 541094 SNPs were analysed. In all cases, run times were feasible, and results precise. The optimal logical combination of classifiers was also determined for all three data sets. Code and data are available from http://bioinformatics.monash.edu.au/downloads/.

**Conclusions:**

The examples demonstrate the methods are suitable for both small and large data sets, applicable to the wide range of bioinformatics classification problems, and robust to dependence between classifiers. In all three test cases, the globally optimal logical combination of the classifiers was found to be their union, according to three out of four ranking criteria. We propose as a general rule of thumb that the union of classifiers will be close to optimal.

## Background

A common problem arising in bioinformatics is to classify experimental results into two categories, according to the presence or absence of some property of interest. Such classification problems are widespread and diverse. For example, in genome-wide association studies (GWAS), genotype data is collected at SNP or other marker loci across the entire genome for a large number of cases and controls (as in [1]), and the marker loci are classified according to whether or not they are associated with the disease under study. Another example is the prediction of protein subcellular localisation, in which predictors such as protein sequence are used to identify to which internal structures, or *organelles*, a protein will be transported after synthesis. A third example is the use of morphological differences to classify cells and tissues, for example to classify whether a cell is cancerous or not, or to determine whether a cell has a parasitic infection.

Despite the diversity of these applications, each can be reduced to binary classification, e.g. disease-associated or non-associated, trafficked or not, infected or parasite-free, etc. Whatever the specific context, it is important to quantify the accuracy of the classifier, in order to assess the level of confidence one should place in the predictions, and so that alternative classifiers can be compared and ranked. Classifiers can be assessed in terms of their *sensitivity* and *specificity*. The sensitivity of a binary classifier is the proportion of positive individuals that are correctly identified as such. Similarly, the specificity is the proportion of negative individuals that are correctly identified.

Ideally, there will be a *gold standard* data set available for evaluating classifiers, comprised of data for which the true classification of each individual is known with certainty, or at least for which there is an accepted best available classification. When a gold standard is available, it is straightforward to estimate the sensitivity and specificity as proportions of the gold standard positives and negatives respectively. However, it can happen that there is no gold standard. For example, if the classifier is a medical diagnostic test such as a swab for swine flu, there may not be any more accurate means of diagnosing the disease, or none that is affordable for a large study. Because such diagnostics are experimental in nature, it is not even possible to simulate data against which to benchmark, and a gold standard for evaluating them may therefore be difficult or impossible to obtain. In bioinformatics the absence of a gold standard can occur because data that can be classified with perfect accuracy is either non-existent or too limited for reliable estimation. It may also be that any gold standard data that was available has already been used to train one or more of the classifiers, rendering the data unsuitable for comparing the classifiers. A concrete example is the analysis of genome-wide association studies, where the data set can include millions of individual SNPs, a negligible proportion of which are known to be associated with the disease. Simulated data can sometimes be generated, but it may not be clear that it is sufficiently realistic. In such cases, it is still possible in principle to evaluate and compare competing classifiers, provided that multiple classifiers are available. In medical diagnostics, this is typically in the order of 2-6 classifiers.

The intuition here is that the extent to which competing classifiers agree or disagree provides information about the reliability of each classifier. In the absence of a gold standard, all that is known is the imperfect binary classifications, which can be organised into a matrix such as shown in Table 1 for *K* classifiers and *N* individuals. Note that the true classification is not known for any individual. However, in broad terms, where two classifiers tend to agree (i.e. have similar columns) our confidence in both of them increases, whereas where two classifiers tend to disagree (i.e. have dissimilar columns), we cannot have high confidence in both. This intuition is given a mathematical expression in the Bayesian models discussed below.

**Table 1:** Data matrix

	**Classifier**			
**Individual**	**1**	**2**	**…**	***K***
1	0	1	…	0
2	1	0	…	1
⋮	⋮	⋮	⡆	⋮
*N*	1	1	…	1

A variety of techniques have been proposed in the medical statistics literature for comparing diagnostic tests in the absence of a gold standard. A spectrum of approaches has been developed to suit specific variations of the problem; for example, some approaches assume log-linear models of errors, as compared to error models that assume normally distributed errors. Approaches also differ in methodology, for example, maximum likelihood approaches compared to Bayesian approaches. Not only do the approaches vary, but the assumptions also vary, with some approaches requiring data from two or more populations with different prevalences of the disease (for example [2]), and others considering tests re-administered to the same individuals at two or more time-points (for example [3]). A review of the diverse approaches and methods is provided by [4]. We focus on the simplest and most common setting in practice, in which *K* binary classifiers are applied to *N* individuals randomly selected from a single population, each at a single time-point. In particular, we consider the Bayesian model of [5,6], and its potential for application to data sets in the bioinformatics domain. This model is described in detail in the first sub-section of the Methods. To the best of our knowledge, the model has not previously been applied in this domain, despite the numerous potential applications. We provide a new, concise and highly efficient implementation in WinBUGS. Our implementation is freely available, applicable to any number of classifiers, and as we demonstrate below, is able to handle genomic-scale data sets.

Once classifiers have been compared, the question naturally arises how to combine them to form a new classifier that is better than any of the constituents. A simple method is to take a consensus, that is, to classify an individual as positive if most of the component classifiers ‘vote’ for a positive classification, and classify an individual as negative otherwise. A weighted consensus, in which the vote of some classifiers counts more than others, is also possible. But what is the optimal way to combine classifiers? This problem has been extensively studied (see [7] for an introduction and [8] for an extended treatment). However, the problem of how to combine classifiers in the absence of gold standard data does not appear to have been studied, and in particular the potential for the model of Joseph *et al.*[5] to solve this problem has not been explored. In the second sub-section of the Methods, we present a new method that estimates the sensitivity and specificity of all logical combinations of classifiers in the absence of a gold standard. The method is implemented in R and again the code is freely available. We note that if no additional covariates are available to distinguish individuals, that is, if the only information we have for each individual is its set of classifications, then logical combinations of the classifiers encompass all possible ways of combining the classifiers, including all weighted voting schemes. The use of additional covariates to better combine classifiers may be a possible extension of the methods proposed here, but is beyond the scope of this paper.

## Results and discussion

The model was loaded into WinBUGS, and run with three test data sets: protein subcellular localisation, to test performance in the presence of a gold standard; swine flu diagnostics, to test performance with a small data set; and classification of SNPs in macular degeneration, to test performance with a large data set. Test data are provided in Additional file 1: Section S2 and on our website.

### Evaluation with gold standard data: classifying protein subcellular localisation

We first evaluated our method relative to a gold standard. Protein sequences were obtained from the Arabidopsis Proteome in FASTA format from the website of [9]: http://bioinfo3.noble.org/AtSubP/. The localisation of these sequences to each of the cytoplasm, chloroplast, golgi body, mitochondrion, nucleus, plasma membrane, and extracellular region is known. The protein sequences were run through three classifiers available from the AtSubP website: the Amino Acid composition-based SVM (AA), Dipeptide composition-based (DP), and N-Center-C Terminal Region-based (NCC) classifiers. These classifiers were converted into binary classifiers by taking one organelle (say, chloroplasts) and treating localisation to that organelle as a positive result, with localisation to a different organelle as a negative result. Results for all seven organelles are presented in Additional file 1: Section S3.1, but in the main text we present only the results for chloroplasts, which were representative (neither best nor worst).

**Figure 1:**
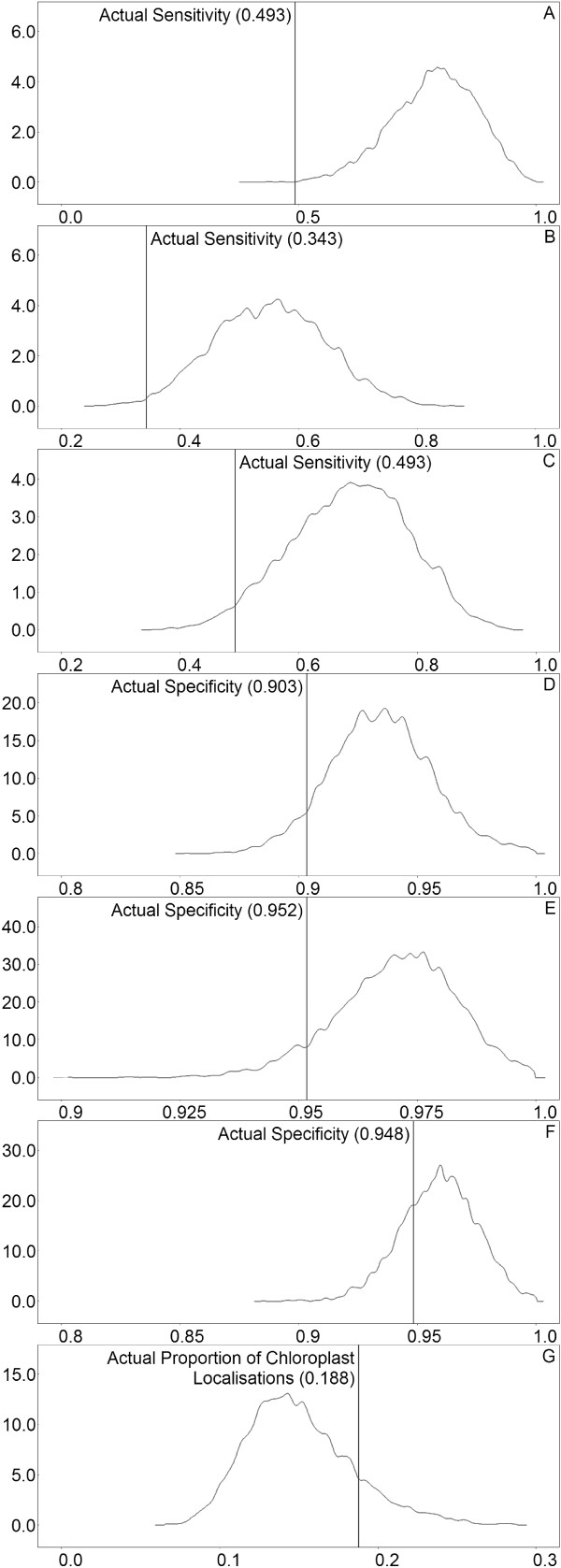
**Protein sub-cellular localisation results.** Density plots of model variables for the chloroplast localisation data. Vertical lines show gold standard sensitivity, specificity or proportion. **A**, **B**, **C**: the sensitivity of the AA , DP and NCC classifiers, respectively. **D**, **E**, **F**: the specificity of the AA , DP and NCC classifiers, respectively. **G**: estimated proportion of proteins localised to chloroplasts.

The output of each classifier was converted to a sequence of 0s and 1s, indicating which proteins were localised to the chloroplast region (1) and which were not (0). For each parameter of the model, summary statistics for its marginal posterior distribution were obtained (Additional file 1: Section S3.2). Density plots (Figure 1) and time series plots (Additional file 1: Section S3.3) were also produced. Sensitivities and specificities for the classifiers were evaluated using the gold standard classification, and these are shown as vertical lines in Figure 1. It should be noted that these gold standard estimates also have standard errors (not shown) because they are based on finite sample sizes.

Our inferred mean posterior sensitivities were typically greater than 2 standard deviations above gold standard estimates, but the latter were nevertheless within the range of values obtained. A similar statement applies to specificities, and the prevalence of chloroplast localisation. Importantly, the classifiers were ranked in the correct order of sensitivity and specificity. That is, Classifiers 2, 3 and 1 had increasing sensitivity and decreasing specificity (see Additional file 1: Section S3.1). These comments apply to all seven organelles, with the caveat that out of the 42 sensitivities and specificities, 5 cases resulted in a tie according to the gold standard. For example, classifiers AA and DP had equal sensitivities when applied to mitochondria. In these cases, the method has not identified a tie, but has otherwise ranked the classifiers correctly.

### Application to a small data set - Swine Flu

We then tested our method on data for the diagnosis of swine flu in patients, where the data set is very small and no gold standard is available. The data contains the diagnosis of *N*=48 individuals for presence or absence of swine flu using *K*=2 different diagnostic tests. The tests are referred to as the *nasopharyngeal aspirate* (NPA) and *nasal swab* (NS). The parameters of the model were each initialised to 0.5 and the model allowed to run for 10000 iterations. Summary statistics and time series plots were obtained for all parameters (Additional file 1: Sections S4.1 and S4.2). There was no discernible burn-in, indicating rapid convergence.

**Figure 2:**
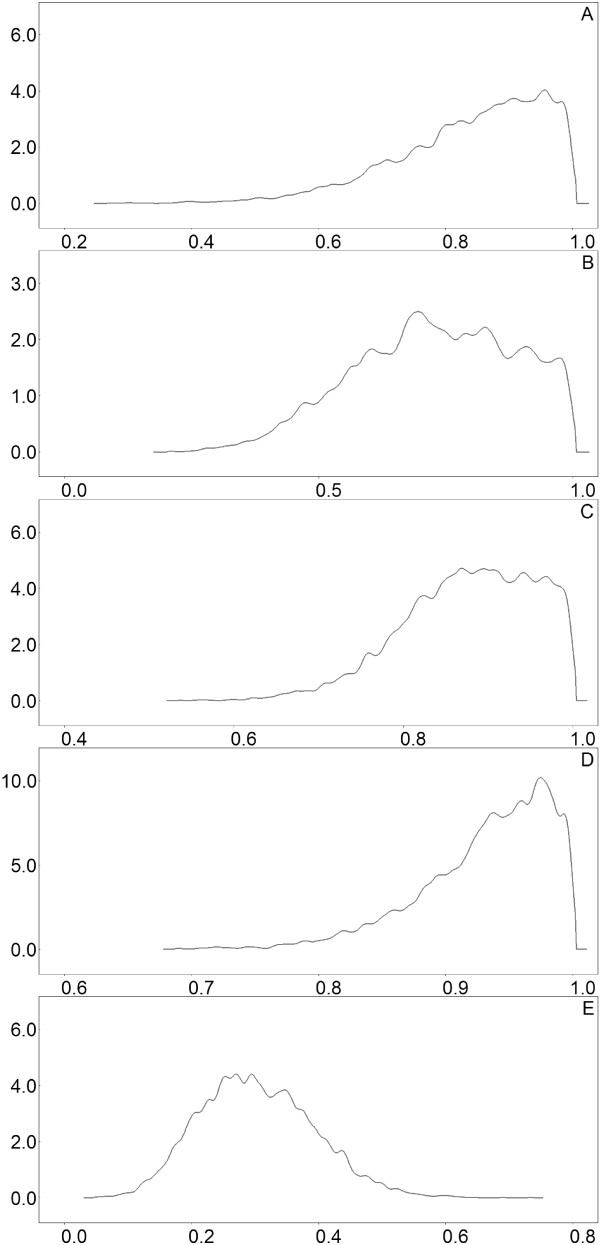
**Swine flu results.** Density plots of model variables for the swine flu data. **A:** Sensitivity of the NPA classifier. **B:** Sensitivity of the NS classifier. **C:** Specificity of the NPA classifier. **D:** Specificity of the NS classifier. **E:** Prevalence of the disease.

Density plots were produced using the last 5000 iterations of the time-series, as shown in Figure 2. The inferred densities exhibited low standard deviations, with an average standard deviation of 0.099 and a maximum of 0.1568, indicating surprisingly good confidence in determining the parameters with a small data set (see Additional file 1: Section S4.1 for means and standard deviations of all parameters). Notice in Figure 2 that the sensitivity of the NPA test (A) is substantially higher than the sensitivity of the NS test (B). However, the specificity of NPA (C) is marginally lower than the specificity of NS (D). On balance, the NPA appears to be the better test, and this conclusion is supported by the ranking criteria that we introduce below (“Inference of the best combination of classifiers”). Note that, in Additional file 1: Section S7.2.3, the NPA test (*C*_1_) scores higher than NS (*C*_2_) according to all four ranking criteria.

### Application to a large data set - SNP classification

To test the method’s performance on a large data set, data from a genome-wide association study was analysed. This study identified SNPs associated with age-related macular degeneration, according to *K*=3 independent classifiers (obtained from [10]). The classifications were produced by running the classifiers on a filtered set of SNPs in the HapMap Phase I+II CEU data (see [10] for details of this filtering procedure). The post-filtered set of SNPs contained *N*=541094 SNPs. The first classifier was PLINK [11], which took as a predictor variable the dosage of the minor allele, and as covariates, the age and gender of each individual. The second classifier was a gene-based method using VEGAS ([12]) and the third classifier was an evaluation of the proportion of significant pairwise interactions between SNPs involving each SNP (as presented in [10]). These methods all assigned p-values to each SNP. It was decided (arbitrarily) to assign approximately the top 1000 SNPs, as classified by each method, to the disease-associated class. To do this, thresholds needed to be set for each classifier. The maximum thresholds that accepted the smallest number of SNPs equal to or greater than 1000 were 0.00195 for PLINK, 0.0019 for the SNP by SNP interaction method and 0.0051 for the gene-based method. These accepted numbers of SNPs were 1003, 1006 and 1310, respectively. For comparison, a second data set with approximately 5000 SNPs was also generated, using thresholds 0.0092, 0.0094 and 0.0232 to obtain 5008, 5362 and 5018 positives respectively.

The parameters of the model were again initialised to 0.5, and the model run for 10000 iterations. Summary statistics were produced for each parameter (Additional file 1: Section S5.1). The last 5000 iterations were used to produce plots of the densities of the sensitivity, specificity and estimated prevalence of disease association (Additional file 1: Section S5.2 and S5.3).

The summary statistics and density plots show smaller standard deviations than the swine flu data, indicating greater confidence in predicting model parameters, which can be attributed to the larger data set. Our method finds that the PLINK classifier had significantly higher sensitivity than the other two classifiers, and slightly higher specificity as well. However, all three had high specificities, as a consequence of classifying such a small proportion of the data as positive.

The difference between the results for the different thresholds is unexpected and informative. Our expectation was that using higher thresholds would increase the sensitivities of all three classifiers and decrease the specificities. Moreover, ideally the method should obtain roughly the same estimate of disease prevalence regardless of the thresholds, since the underlying population is the same. Instead, using the higher thresholds resulted in lower estimates of both sensitivity and specificity, but only slightly (compare tables and density plots in Additional file 1: Section S5). A more dramatic change is that the estimate of disease prevalence increased approximately six-fold. This unexpected behaviour may be indicative of conditional dependence between the classifiers.

Notably however, *the ranking of the three classifiers by sensitivity or by specificity remains the same - with PLINK significantly outperforming the other classifiers on both measures*. This observation underscores our major point that the ranking of classifiers is in general robust to violation of the assumption of conditional independence.

### Inference of the best combination of classifiers

The R-script described in the Methods was used to invoke the WinBUGS model from R (using R2WinBUGS), and the model was rerun for all three test cases for a burn-in of 1000 iterations. Then, for each case the model was run for a further 1000 iterations, and at every iteration an estimate of the sensitivity and specificity was calculated for all possible logical combinations of the classifiers. Only the last 500 iterations were used for the following analyses.

To determine which logical combination of the classifiers performed best, we applied the four ranking criteria based on the (1) product, (2) sum of squares, (3) sum of absolute values, and (4) minimum of the sensitivity and specificity. Summary statistics for sensitivities and specificities of all logical combinations of the swine flu classifiers, and selected simple combinations of the chloroplast localisation and SNP classifiers, are shown in Tables 2, 3 and 4. For all three test cases, ranking criteria 1-3 (product, sum of squares, and sum of absolute values) identified the union of all classifiers as the best combination. For the SNP data, ranking criterion 4 (minimum of the sensitivity and specificity) also inferred that the union of all classifiers was best, while for the subcellular localisation and swine flu data, the best combination was a union of all but one of the classifiers. The better performance of a union of classifiers is due to higher sensitivity at the expense of lower specificity, as the union of all classifiers necessarily has higher sensitivity and lower specificity than any union of a subset of the classifiers.

**Table 2:** Sensitivities and specificities of the chloroplast localisation classifier combinations

		**Sensitivity**			**Specificity**						
**2**^***K***^**-bit code**	**Combination**	**Mean**	**Median**	**SD**	**Mean**	**Median**	**SD**				
2	*C*_1_∧*C*_2_∧*C*_3_	0.311	0.307	0.094	1.000	1.000	0.000
4	*C*_2_∧*C*_3_	0.394	0.384	0.096	0.999	0.999	0.001
6	*C*_1_∧*C*_3_	0.553	0.544	0.108	0.997	0.997	0.001
18	*C*_1_∧*C*_2_	0.436	0.433	0.101	0.998	0.998	0.001
24	At least two classifiers	0.762	0.772	0.089	0.994	0.995	0.003
64	*C*_2_∨*C*_3_	0.867	0.870	0.058	0.932	0.933	0.021
96^*†*^	*C*_1_∨*C*_3_	0.934	0.939	0.037	0.894	0.895	0.025
120	*C*_1_∨*C*_2_	0.900	0.910	0.053	0.904	0.907	0.025
128^*‡*^	*C*_1_∨*C*_2_∨*C*_3_	0.969	0.975	0.021	0.868	0.869	0.029

SD: Standard Deviation.

^‡^Optimal combination using ranking criteria 1, 2 and 3.

^†^Optimal combination using ranking criterion 4.

**Table 3:** Sensitivities and specificities of the swine flu classifier combinations

		**Sensitivity**			**Specificity**		
2^***K***^**-bit code**	**Combination**	**Mean**	**Median**	**SD**	**Mean**	**Median**	**SD**
1	All results are negative	0.000	0.000	0.000	1.000	1.000	0.000
2	*C*_1_∧*C*_2_	0.626	0.633	0.162	0.991	0.994	0.010
3	¬*C*_1_∧*C*_2_	0.116	0.101	0.088	0.938	0.947	0.047
4	*C*_2_	0.742	0.760	0.152	0.928	0.939	0.054
5	*C*_1_∧¬*C*_2_	0.214	0.201	0.127	0.879	0.885	0.072
6^*†*^	*C*_1_	0.840	0.859	0.119	0.870	0.875	0.078
7	(¬*C*_1_∧*C*_2_)∨(*C*_1_∧¬*C*_2_)	0.330	0.340	0.124	0.817	0.821	0.079
8^*‡*^	*C*_1_∨*C*_2_	0.957	0.974	0.050	0.808	0.813	0.087
9	¬(*C*_1_∨*C*_2_)	0.043	0.026	0.050	0.192	0.187	0.087
10	(*C*_1_∧*C*_2_)∨¬(*C*_1_∨*C*_2_)	0.670	0.660	0.124	0.183	0.179	0.079
11	¬*C*_1_	0.160	0.141	0.119	0.130	0.125	0.078
12	¬*C*_1_∨*C*_2_	0.786	0.799	0.127	0.121	0.115	0.072
13	¬*C*_2_	0.258	0.240	0.152	0.072	0.061	0.054
14	*C*_1_∨¬*C*_2_	0.884	0.899	0.088	0.062	0.053	0.047
15	¬(*C*_1_∧*C*_2_)	0.374	0.367	0.162	0.009	0.006	0.010
16	All results are positive	1.000	1.000	0.000	0.000	0.000	0.000

SD: Standard Deviation.

^‡^Optimal combination using ranking criteria 1, 2 and 3.

^†^Optimal combination using ranking criterion 4.

**Table 4:** Sensitivities and specificities of the SNP classifier combinations

**∼ 1000 positives**		**Sensitivity**			**Specificity**		
**2**^***K***^**-bit code**	**Combination**	**centerMean**	**Median**	**SD**	**Mean**	**Median**	**SD**
2	*C*_1_∧*C*_2_∧*C*_3_	0.083	0.082	0.017	1.000	1.000	0.0000
4	*C*_2_∧*C*_3_	0.098	0.097	0.018	1.000	1.000	0.0000
6	*C*_1_∧*C*_3_	0.144	0.143	0.020	1.000	1.000	0.0000
18	*C*_1_∧*C*_2_	0.483	0.481	0.053	1.000	1.000	0.0000
24	At least two classifiers	0.559	0.558	0.053	1.000	1.000	0.0000
64	*C*_2_∨*C*_3_	0.645	0.647	0.047	0.997	0.997	0.0001
96	*C*_1_∨*C*_3_	0.869	0.869	0.035	0.997	0.997	0.0001
120	*C*_1_∨*C*_2_	0.932	0.933	0.021	0.998	0.998	0.0001
128^*‡*^	*C*_1_∨*C*_2_∨*C*_3_	0.943	0.944	0.018	0.996	0.996	0.0002
∼ 5000 positives		Sensitivity			Specificity		
2^*K*^-bit code	Combination	Mean	Median	SD		Mean	Median	SD
2	*C*_1_∧*C*_2_∧*C*_3_	0.065	0.066	0.007		1.000	1.000	0.0000
4	*C*_2_∧*C*_3_	0.079	0.079	0.007		1.000	1.000	0.0000
6	*C*_1_∧*C*_3_	0.123	0.123	0.008		1.000	1.000	0.0000
18	*C*_1_∧*C*_2_	0.439	0.440	0.025		1.000	1.000	0.0000
24	At least two classifiers	0.510	0.511	0.026		1.000	1.000	0.0000
64	*C*_2_∨*C*_3_	0.601	0.601	0.024		0.987	0.987	0.0002
96	*C*_1_∨*C*_3_	0.850	0.852	0.018		0.991	0.991	0.0004
120	*C*_1_∨*C*_2_	0.918	0.919	0.011		0.994	0.994	0.0004
128^*‡*^	*C*_1_∨*C*_2_∨*C*_3_	0.930	0.931	0.010		0.986	0.986	0.0004

SD: Standard Deviation.

^‡^Optimal combination using ranking criteria 1, 2, 3 and 4.

For the two cases with <500 data points (swine flu and subcellular localisation), the optimal logical combination was the same in up to 0.526 iterations of the Gibbs sampler. However, for the SNP data with more than 500000 data points, the optimal classifier was the same at every iteration. This is expected, as more data should increase the confidence with which the optimal classifier can be identified.

Posterior density plots for the sensitivity and specificity of all possible logical combinations of the swine flu classifiers are presented in Additional file 1: Section S7.2, and for selected logical combinations of the subcellular localisation classifiers in Additional file 1: Section S7.1. Additional file 1: Sections S7.1 to S7.3 also contain summary statistics for the four ranking criteria in each case, generated using the R code in Additional file 1: Section S6.2. A curious anomaly is observed for the swine flu data: Ranking Method 4 identified the NPA classifier (*C*_1_) as best in a majority of MCMC iterations, yet the average of the Method 4 score is slightly higher for the union of the NPA and NS classifiers (*C*_1_∨*C*_2_). We note that the standard deviations of the ranking scores are quite large relative to the differences between ranking scores, which may suggest that combinations of classifiers other than that identified as ‘best’ remain plausible candidates. Nevertheless it is clear that the union of all classifiers ranks well if not best for all data sets and any ranking criterion examined here.

### Run times

Run times for the various data sets are shown in Table 5. Times for the 10000 iteration runs for the chloroplast localisation, swine flu, and SNP data are in the column headed ‘WinBUGS’. The times were approximately the same for the swine flu and chloroplast data sets, despite the greater number of data points and extra classifier in the latter. The SNP data set was >1500 times larger and as expected the run time was much greater.

**Table 5:** Run times

**Data**	**No. Subjects**	**WinBUGS**	**WinBUGS(R)**	**R**
Swine Flu	48	625 s	0s	1s
Chloroplasts	357	624 s	1s	12s
SNP	541094	8 hrs	2917s	12s

The run times of the 2000 iteration runs from the previous sub-section included an R component and a WinBUGS (called from R) component, shown in the last two columns. WinBUGS apparently runs faster when called from R. Although the R combination algorithm (second sub-section of the Methods) is $O(2^{2^{K}})$, the main time cost of the genomic scale SNP runs (where *K*=3) is in the run time of the WinBUGS comparison algorithm (first sub-section of the Methods), which is only linear in *K*.

## Conclusions

The method presented in this paper addresses two significant problems with ubiquitous applications in bioinformatics: comparing binary classifiers in the absence of a gold standard, and identifying the optimal logical combination of such classifiers. Using Bayesian models developed for evaluating medical diagnostic tests, we present the first applications of these models in the bioinformatics domain and demonstrate their feasibility and utility for comparing classifiers on genomic scale data sets. A new, concise and highly efficient implementation of these models was developed in WinBUGS, and is the first freely available implementation applicable to an arbitrary number of classifiers. To identify the optimal logical combination of classifiers, we developed an entirely new algorithm and again demonstrated its feasibility for genomic scale data sets. The algorithm is the first to employ the above-mentioned Bayesian models to evaluate logical combinations of classifiers and indeed apparently the first to systematically evaluate all logical combinations. It is implemented in R and is freely available. The algorithm is $O(2^{2^{K}})$ in the number of classifiers *K*, and thus further research is required for large *K*.

The methods were evaluated on a protein subcellular localisation data set for which a gold-standard data set was available for the purpose of comparison. Some discrepancy in the estimates of sensitivity and specificity was expected because a key assumption of the model - conditional independence of the classifiers - is often violated in practice. However, we found that the discrepancy was in most cases small and more importantly, that the method was able to correctly rank the classifiers.

In all of our examples, a simple union of the classifiers was found to be optimal according to three out of four alternative ranking criteria (and in some cases also by the fourth). While this finding is unlikely to be general, we propose as a rule of thumb that the union of classifiers is likely to be close to the optimal logical combination.

## Methods

### Estimating sensitivities and specificities

In this section, we describe a Bayesian model that combines features of the models of [5,6]. As with any Bayesian model, one first defines a likelihood model, that is the probability of the data given some values of the parameters. One then defines prior probabilities and applies Bayes’ rule to obtain posterior probabilities for the parameters, given the data. Finally, one can sample from this posterior distribution using Markov chain Monte Carlo (MCMC). Such samples can then be used to construct marginal posterior densities, as we do in the Results and discussion section.

Following [5], our model considers the true positive (sensitivity) and false positive (one minus specificity) rates of each classifier as parameters to be estimated. Following [6], and in contrast to [5], we explicitly estimate the latent true classifications for each individual in each iteration of the sampler. This enables a simple implementation of the model (less than 10 lines of WinBUGS code) that is applicable to any number of classifiers. In contrast, the implementation of [5] must be specifically encoded for a fixed number of classifiers, and becomes increasingly unwieldy as the number of classifiers increases.

**Figure 3:**
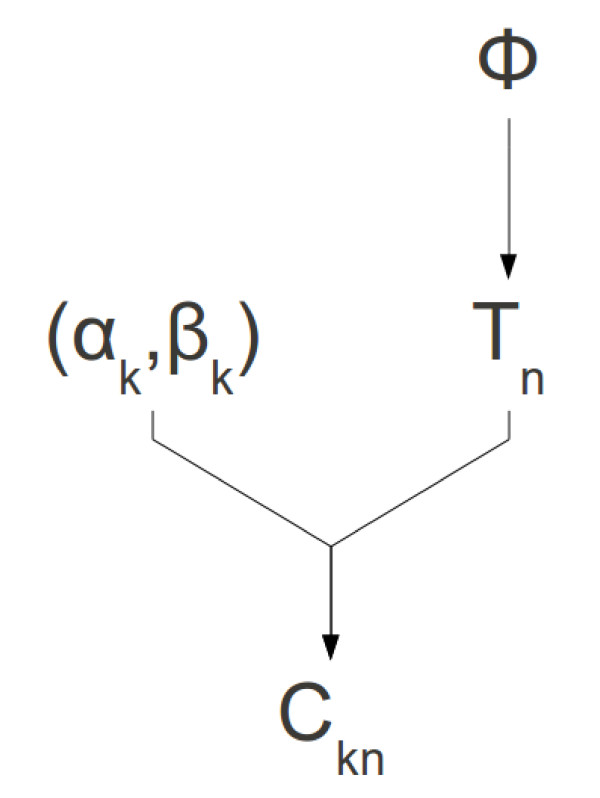
**Conditional dependencies of the model.** The dependencies of parameters in the model. *ϕ*is the proportion of the population that is positive for the feature of interest, *T*_*n*_is the true classification of individual *n*, *α*_*k*_and *β*_*k*_are the probabilities of a true positive and a false positive (respectively) for classifier *k*, and *C*_*kn*_is the classification of individual *n* according to classifier *k*.

Figure 3 shows the conditional dependencies of the model, for an arbitrary number of individuals *N* and classifiers *K*. Let *C*_*kn*_ be the outcome of Classifier *k* for each individual *n*, with *C*_*kn*_=1 indicating a positive result and *C*_*kn*_=0 indicating a negative result. These outcomes are modeled as independent Bernoulli trials, conditional on the true classification for each individual (that is, the classifiers are *conditionally independent*). Let the true classification for individual *n* be *T*_*n*_ and let *α*_*k*_ and *β*_*k*_ denote the true positive and false positive rates of Classifier *k*, respectively. Hence:

(1) $$P(C_{\mathrm{kn}}|T_{n},\alpha_{k},\beta_{k})=\left\{\begin{array}{cc} \alpha_{k} & C_{\mathrm{kn}}=1,T_{n}=1\\ 1-\alpha_{k} & C_{\mathrm{kn}}=0,T_{n}=1\\ \beta_{k} & C_{\mathrm{kn}}=1,T_{n}=0\\ 1-\beta_{k} & C_{\mathrm{kn}}=0,T_{n}=0 \end{array}\right)$$

Let the proportion of the population under study that has the feature of interest be *ϕ*and further suppose that the *N* individuals were selected uniformly and randomly from this population, without replacement. This is mathematically equivalent to assuming that the true classifications *T*_*n*_are the outcomes of *N* independent Bernoulli trials with probability *ϕ* of a positive individual. The model thus has *N* + 2*K* + 1 parameters.

This part of the model would be the same regardless of whether one adopted a Bayesian approach. What makes a model Bayesian is the specification of prior probabilities for the parameters of interest. Here we assign uniform priors on the interval [0,1] for each of the parameters *ϕ*, *α*_*k*_ and *β*_*k*_. This means that, prior to observing the data, all possible values between 0 and 1 are considered to be equally likely. In addition, the model applies the constraints *α*_*k*_≥*β*_*k*_, since a classifier would be better discarded if it is more likely to classify an individual as positive when that individual is actually negative. Note this inequality introduces dependence between *α*_*k*_ and *β*_*k*_ for a single classifier, but does not violate the conditional independence of distinct classifiers.

Having defined the likelihood and prior probabilities, the model is straightforward to implement with the freely available Bayesian software package WinBUGS ([13]). Perhaps surprisingly, it is not necessary to use Bayes’ rule to determine the posterior probabilities analytically, as WinBUGS automates this step. Nor is it necessary to work out how to sample from the posterior distribution: WinBUGS automates this as well. WinBUGS uses an MCMC method - the well-known Gibbs Sampler - which cycles through the parameters, updating each by drawing from the posterior distribution for that parameter, conditional on all other parameters remaining constant. Code for our implementation is included as Additional file 1: Section S1, and on our website.

### Inferring the best combination of classifiers

Another important goal is to decide how best to combine a collection of classifiers using the logical operators AND (∧), OR (∨) and NOT (¬). As applied to classifiers, the complement (¬X) classifies an individual as positive if and only if *X* classifies that individual as negative. The intersection (*X*∧*Y*) classifies an individual as positive if and only if both *X* and *Y * classify that individual as positive. The union (*X*∨*Y*) classifies an individual as positive if and only if either *X* or *Y * classifies that individual as positive.

The sensitivity and specificity of any logical combination of classifiers can be calculated from the sensitivities and specificities of the constituent classifiers, if one assumes conditional independence. Here we propose to evaluate the sensitivity and specificity of every possible logical combination, using estimates for the constituents obtained using the method of the previous sub-section. This can therefore be done in the absence of a gold standard.

One problem is that there are infinitely many semantically correct ways to arrange the symbols $C_{1},\ldots ,C_{K},\wedge ,\vee ,\neg ,$ ( and ) to form a logical combination, because in principle there is no limit to the number of times each classifier can appear. A second problem is that such expressions are not unique, in the sense that equivalent classifiers can be obtained by different expressions involving the above symbols. To solve both of these problems, we express logical combinations in a canonical form, of which there are a finite number. We also define an ordering of these canonical forms, so that they can be evaluated systematically. The canonical form is obtained by noting that any logical combination of the classifiers *C*_1_,…,*C*_*K*_ can be reformulated as a disjoint union of intersections of the form (*X*_1_∧*X*_2_∧…∧*X*_*K*_), where each *X*_*k*_is either *C*_*k*_or $\neg C_{k}$, and all classifiers (or their complements) are present in each such intersection.

The sensitivity and specificity of a logical combination of classifiers can be built up from the following primitive rules applied to the canonical forms. First, the sensitivity (SENS) and specificity (SPEC) of ¬X are given by 1−SENS(*X*) and 1−SPEC(*X*) respectively. Let *T* denote the set of all positive individuals. Using conditional independence, the sensitivity of an intersection is given by:

(2) $$\begin{array}{lll} \mathrm{SENS}(X\wedge Y) & \;=\;\mathrm{Pr}(X\cap Y|T)\; & \;\\ & =\;\mathrm{Pr}(X|T)\times \mathrm{Pr}(Y|T)\; & \;\\ & =\;\mathrm{SENS}(X)\times \mathrm{SENS}(Y)\; & \; \end{array}$$

and the specificity by:

(3) $$\begin{array}{lll} \mathrm{SPEC}(X\wedge Y) & \;=\;\mathrm{Pr}({\left(X\cap Y\right)}^{c}|T^{c})\; & \;\\ & =\;\mathrm{Pr}(X^{c}\cup Y^{c}|T^{c})\; & \;\\ & =\;\mathrm{Pr}(X^{c}|T^{c})+\mathrm{Pr}(Y^{c}|T^{c})-\mathrm{Pr}(X^{c}|T^{c})\; & \;\\ & \;\times \mathrm{Pr}(Y^{c}|T^{c})\; & \;\\ & =\;\mathrm{SPEC}(X)+\mathrm{SPEC}(Y)-\mathrm{SPEC}(X)\; & \;\\ & \;\times \mathrm{SPEC}(Y).\; & \; \end{array}$$

Here we have freely used *X* to denote both a classifier and the set of individuals classified as positive by that classifier.

To systematically evaluate the sensitivity and specificity of all intersections of the form (*X*_1_∧…∧*X*_*K*_) mentioned above, we assign a *K*-bit code to each such intersection. Where *X*_*k*_=*C*_*k*_, the *k*th bit is set to 0, and where $X_{k}=\neg C_{k}$, the *k*th bit is set to 1 (with bits numbered right to left). For example, given three classifiers, the *k*-bit code for $C_{1}\wedge \neg C_{2}\wedge \neg C_{3}$ would be 110. There are thus 2^*K*^such intersections, and we compute their sensitivities and specificities in the order indicated by their bit codes. Let the sensitivity and specificity of this *K*-bit code for the *j*th such intersection be SENS_K(*j*) and SPEC_K(*j*), respectively, where *j* runs from 0 to 2^*K*^−1.

It remains to compute the sensitivity and specificity of any disjoint union of these 2^*K*^intersections. A disjoint union of classifiers has sensitivity:

(4) $$\begin{array}{lll} \mathrm{SENS}(X\vee Y) & \;=\;\mathrm{Pr}(X\cup Y|T)\; & \;\\ & =\;\mathrm{Pr}(X|T)+\mathrm{Pr}(Y|T)\; & \;\\ & =\;\mathrm{SENS}(X)+\mathrm{SENS}(Y)\; & \; \end{array}$$

and specificity:

(5) $$\begin{array}{lll} \mathrm{SPEC}(X\vee Y) & \;=\;\mathrm{Pr}({\left(X\cup Y\right)}^{c}|T^{c})\; & \;\\ & =\;\mathrm{Pr}(X^{c}\cap Y^{c}|T^{c})\; & \;\\ & =\;\mathrm{Pr}(X^{c}|T^{c})+\mathrm{Pr}(Y^{c}|T^{c})-1\; & \;\\ & =\;\mathrm{SPEC}(X)+\mathrm{SPEC}(Y)-1.\; & \; \end{array}$$

These two rules suffice to calculate the sensitivity and specificity of any disjoint union, but again the 2^*K*^intersections must be processed systematically. We assign a second 2^*K*^-bit code to each disjoint union, with bit *j* set to 1 or 0 according to whether the intersection *j* is included or excluded from the union (with bits numbered right to left). For example, given two classifiers, the 2^*K*^-bit code 0011 represents $\left(C_{1}\wedge C_{2})\vee (\neg C_{1}\wedge C_{2}\right)$. Hence there are $2^{2^{K}}$ logical combinations in all, and we again evaluate sensitivity and specificity in the order corresponding to their bit codes. For disjoint union *m*, we denote the sensitivity and specificity of this 2^*K*^-bit code by SENS_2K(*m*) and SPEC_2K(*m*) respectively. In practice, this is implemented using the formulae:

(6) $$\mathrm{SENS}\text{\_}2K(m)=\overset{2^{K}-1}{\underset{j=0}{\sum }}g_{j}(m)$$

and

(7) $$\mathrm{SPEC}\text{\_}2K(m)=\left(\overset{2^{K}-1}{\underset{j=0}{\sum }}h_{j}(m)\right)+1,$$

where:

(8) $$g_{j}(m)=\left\{\begin{array}{cc} \mathrm{SENS}\text{\_}K(j) & \;\;\text{if union}m\text{includes}\\ \;\;\text{intersection}j\\ 0 & \;\;\text{if union}m\text{does not}\\ \;\;\text{include intersection}j \end{array}\right)$$

and

(9) $$h_{j}(m)=\left\{\begin{array}{cc} \mathrm{SPEC}\text{\_}K(j)-1 & \;\;\text{if union}m\text{includes}\\ \;\;\text{intersection}j\\ 0 & \;\;\text{if union}m\text{does not}\\ \;\;\text{include intersection}j \end{array}\right)$$

We use the model from the previous sub-section to determine the optimal logical combination as follows. Each iteration of the Gibbs Sampler produces an estimate of the sensitivity and specificity for each of the *K* classifiers. Using these estimates, we calculate the corresponding sensitivity and specificity of every possible logical combination of the classifiers, using the systematic procedure described in the preceding paragraphs. We then select the optimal logical combination according to one of four suitable ranking criteria: the product, sum of squares, sum of absolute values, and minimum of the sensitivity and specificity.

The optimal logical combination, thus determined, may differ from one iteration of the Gibbs sampler to the next. We therefore estimate the probability that any given logical combination is optimal as the proportion of Gibbs sampler iterations in which that combination was optimal. The overall best combination is then the one that is found to be optimal in the greatest proportion of iterations.

Note that this method exhaustively enumerates all ways in which classifiers can be combined, if all that is known about each individual is the classifications (i.e. only data of the form illustrated in Table 1 are available, and there are no additional covariates that can be used to distinguish between individuals with identical rows). This includes all possible weighted voting schemes. The globally optimal combination is therefore identified.

Note that enumeration of all possible logical combinations of classifiers necessarily requires computational time $O(2^{2^{K}})$, and thus rapidly becomes infeasible as the number of classifiers increases. In practice, the method is only feasible for *K*≤5 classifiers. Further research into methods capable of handling a larger number of classifiers is needed.

R code implementing this method is available in Additional file 1: Section S6.1, and from our website. It is also interesting to construct posterior distributions of the sensitivities and specificities for each possible logical combination, as we illustrate in the Results and discussion.

### System and Implementation

The model was implemented in the freely available Microsoft^*®*^ Windows-based Bayesian Analysis software, WinBUGS v1.7 [13], on a Dell^TM^ Optiplex^TM^ 980 computer with a quad core 3.33 GHz Intel^®;^ Core^TM^ i5 processor. To combine classifiers, output from the WinBUGS runs was loaded into the statistical package R [14], using the R2WinBUGS library [15]. Calculation of the sensitivities and specificities of all logical combinations of classifiers was performed using R (code available at our website).

## Competing interests

The authors declare that they have no competing interests.

## Author’s contributions

JMK conceived the idea of the methods, algorithms and applications, developed them on paper, wrote the WinBUGS code, and worked extensively on all parts of the text; CMD wrote the R code, ran all of the computational experiments, produced all tables and figures, and wrote the initial draft of the text; SEB searched the literature and the internet for relevant previous work and suitable data sets, in particular identifying the sub-cellular localisation data as suitable for verifying our methods, and worked extensively on all parts of the text. All authors read and approved the final manuscript.

## Supplementary Material

Additional file 1 **Supplementary Material.** All supplementary material is contained in the file ‘comparing-binary-classifiers-180712-bmc-supp-v3.doc’. Click here for file
