# Supplementary material for: A Bayesian method for comparing and combining binary classifiers in the absence of a gold standard
Source: BMC Bioinformatics. 2012 Jul 27;13:179. doi: 10.1186/1471-2105-13-179 (PMC3473310; doi:10.1186/1471-2105-13-179)
Supplement: Additional file 1 — Supplementary Material. All supplementary material is contained in the file ‘comparing-binary-classifiers-180712-bmc-supp-v3.doc’. [file 1471-2105-13-179-S1.doc]

**Supplementary Data to Bayesian Methods for Comparing and Combining Binary Classifiers in the Absence of a Gold Standard**

Jonathan M Keith^1,*^, Christian M Davey^1^ and Sarah E Boyd^1^

^1^School of Mathematical Sciences, Monash University, Victoria, Australia

**S0. Licence for code provided in this document**

GNU GENERAL PUBLIC LICENSE

Version 2, June 1991

Copyright (C) 1989, 1991 Free Software Foundation, Inc.

51 Franklin Street, Fifth Floor, Boston, MA 02110-1301, USA

Everyone is permitted to copy and distribute verbatim copies

of this license document, but changing it is not allowed.

Preamble

The licenses for most software are designed to take away your freedom to share and change it. By contrast, the GNU General Public License is intended to guarantee your freedom to share and change free software--to make sure the software is free for all its users. This General Public License applies to most of the Free Software Foundation's software and to any other program whose authors commit to using it. (Some other Free Software Foundation software is covered by the GNU Lesser General Public License instead.) You can apply it to your programs, too.

When we speak of free software, we are referring to freedom, not price. Our General Public Licenses are designed to make sure that you have the freedom to distribute copies of free software (and charge for this service if you wish), that you receive source code or can get it if you want it, that you can change the software or use pieces of it in new free programs; and that you know you can do these things.

To protect your rights, we need to make restrictions that forbid anyone to deny you these rights or to ask you to surrender the rights. These restrictions translate to certain responsibilities for you if you distribute copies of the software, or if you modify it.

For example, if you distribute copies of such a program, whether gratis or for a fee, you must give the recipients all the rights that you have. You must make sure that they, too, receive or can get the source code. And you must show them these terms so they know their rights.

We protect your rights with two steps: (1) copyright the software, and (2) offer you this license which gives you legal permission to copy, distribute and/or modify the software.

Also, for each author's protection and ours, we want to make certain that everyone understands that there is no warranty for this free software. If the software is modified by someone else and passed on, we want its recipients to know that what they have is not the original, so that any problems introduced by others will not reflect on the original authors' reputations.

Finally, any free program is threatened constantly by software patents. We wish to avoid the danger that redistributors of a free program will individually obtain patent licenses, in effect making the program proprietary. To prevent this, we have made it clear that any patent must be licensed for everyone's free use or not licensed at all.

The precise terms and conditions for copying, distribution and modification follow.

TERMS AND CONDITIONS FOR COPYING, DISTRIBUTION AND MODIFICATION

0. This License applies to any program or other work which contains a notice placed by the copyright holder saying it may be distributed under the terms of this General Public License. The "Program", below, refers to any such program or work, and a "work based on the Program" means either the Program or any derivative work under copyright law: that is to say, a work containing the Program or a portion of it, either verbatim or with modifications and/or translated into another language. (Hereinafter, translation is included without limitation in the term "modification".) Each licensee is addressed as "you".

Activities other than copying, distribution and modification are not covered by this License; they are outside its scope. The act of running the Program is not restricted, and the output from the Program is covered only if its contents constitute a work based on the Program (independent of having been made by running the Program). Whether that is true depends on what the Program does.

1. You may copy and distribute verbatim copies of the Program's source code as you receive it, in any medium, provided that you conspicuously and appropriately publish on each copy an appropriate copyright notice and disclaimer of warranty; keep intact all the notices that refer to this License and to the absence of any warranty; and give any other recipients of the Program a copy of this License along with the Program.

You may charge a fee for the physical act of transferring a copy, and you may at your option offer warranty protection in exchange for a fee.

2. You may modify your copy or copies of the Program or any portion of it, thus forming a work based on the Program, and copy and distribute such modifications or work under the terms of Section 1 above, provided that you also meet all of these conditions:

a) You must cause the modified files to carry prominent notices stating that you changed the files and the date of any change.

b) You must cause any work that you distribute or publish, that in whole or in part contains or is derived from the Program or any part thereof, to be licensed as a whole at no charge to all third parties under the terms of this License.

c) If the modified program normally reads commands interactively when run, you must cause it, when started running for such interactive use in the most ordinary way, to print or display an announcement including an appropriate copyright notice and a notice that there is no warranty (or else, saying that you provide a warranty) and that users may redistribute the program under these conditions, and telling the user how to view a copy of this License. (Exception: if the Program itself is interactive but does not normally print such an announcement, your work based on the Program is not required to print an announcement.)

These requirements apply to the modified work as a whole. If identifiable sections of that work are not derived from the Program, and can be reasonably considered independent and separate works in themselves, then this License, and its terms, do not apply to those sections when you distribute them as separate works. But when you distribute the same sections as part of a whole which is a work based on the Program, the distribution of the whole must be on the terms of this License, whose permissions for other licensees extend to the entire whole, and thus to each and every part regardless of who wrote it.

Thus, it is not the intent of this section to claim rights or contest your rights to work written entirely by you; rather, the intent is to exercise the right to control the distribution of derivative or collective works based on the Program.

In addition, mere aggregation of another work not based on the Program with the Program (or with a work based on the Program) on a volume of a storage or distribution medium does not bring the other work under the scope of this License.

3. You may copy and distribute the Program (or a work based on it, under Section 2) in object code or executable form under the terms of Sections 1 and 2 above provided that you also do one of the following:

a) Accompany it with the complete corresponding machine-readable source code, which must be distributed under the terms of Sections 1 and 2 above on a medium customarily used for software interchange; or,

b) Accompany it with a written offer, valid for at least three years, to give any third party, for a charge no more than your cost of physically performing source distribution, a complete machine-readable copy of the corresponding source code, to be distributed under the terms of Sections 1 and 2 above on a medium customarily used for software interchange; or,

c) Accompany it with the information you received as to the offer to distribute corresponding source code. (This alternative is allowed only for noncommercial distribution and only if you received the program in object code or executable form with such an offer, in accord with Subsection b above.)

The source code for a work means the preferred form of the work for making modifications to it. For an executable work, complete source code means all the source code for all modules it contains, plus any associated interface definition files, plus the scripts used to control compilation and installation of the executable. However, as a special exception, the source code distributed need not include anything that is normally distributed (in either source or binary form) with the major components (compiler, kernel, and so on) of the operating system on which the executable runs, unless that component itself accompanies the executable.

If distribution of executable or object code is made by offering access to copy from a designated place, then offering equivalent access to copy the source code from the same place counts as distribution of the source code, even though third parties are not compelled to copy the source along with the object code.

4. You may not copy, modify, sublicense, or distribute the Program except as expressly provided under this License. Any attempt otherwise to copy, modify, sublicense or distribute the Program is void, and will automatically terminate your rights under this License. However, parties who have received copies, or rights, from you under this License will not have their licenses terminated so long as such parties remain in full compliance.

5. You are not required to accept this License, since you have not signed it. However, nothing else grants you permission to modify or distribute the Program or its derivative works. These actions are prohibited by law if you do not accept this License. Therefore, by modifying or distributing the Program (or any work based on the Program), you indicate your acceptance of this License to do so, and all its terms and conditions for copying, distributing or modifying the Program or works based on it.

6. Each time you redistribute the Program (or any work based on the Program), the recipient automatically receives a license from the original licensor to copy, distribute or modify the Program subject to these terms and conditions. You may not impose any further restrictions on the recipients' exercise of the rights granted herein. You are not responsible for enforcing compliance by third parties to this License.

7. If, as a consequence of a court judgment or allegation of patent infringement or for any other reason (not limited to patent issues), conditions are imposed on you (whether by court order, agreement or otherwise) that contradict the conditions of this License, they do not excuse you from the conditions of this License. If you cannot distribute so as to satisfy simultaneously your obligations under this License and any other pertinent obligations, then as a consequence you may not distribute the Program at all. For example, if a patent license would not permit royalty-free redistribution of the Program by all those who receive copies directly or indirectly through you, then the only way you could satisfy both it and this License would be to refrain entirely from distribution of the Program.

If any portion of this section is held invalid or unenforceable under any particular circumstance, the balance of the section is intended to apply and the section as a whole is intended to apply in other circumstances.

It is not the purpose of this section to induce you to infringe any patents or other property right claims or to contest validity of any such claims; this section has the sole purpose of protecting the integrity of the free software distribution system, which is implemented by public license practices. Many people have made generous contributions to the wide range of software distributed through that system in reliance on consistent application of that system; it is up to the author/donor to decide if he or she is willing to distribute software through any other system and a licensee cannot impose that choice.

This section is intended to make thoroughly clear what is believed to be a consequence of the rest of this License.

8. If the distribution and/or use of the Program is restricted in certain countries either by patents or by copyrighted interfaces, the original copyright holder who places the Program under this License may add an explicit geographical distribution limitation excluding those countries, so that distribution is permitted only in or among countries not thus excluded. In such case, this License incorporates the limitation as if written in the body of this License.

9. The Free Software Foundation may publish revised and/or new versions of the General Public License from time to time. Such new versions will be similar in spirit to the present version, but may differ in detail to address new problems or concerns.

Each version is given a distinguishing version number. If the Program specifies a version number of this License which applies to it and "any later version", you have the option of following the terms and conditions either of that version or of any later version published by the Free Software Foundation. If the Program does not specify a version number of this License, you may choose any version ever published by the Free Software Foundation.

10. If you wish to incorporate parts of the Program into other free programs whose distribution conditions are different, write to the author to ask for permission. For software which is copyrighted by the Free Software Foundation, write to the Free Software Foundation; we sometimes make exceptions for this. Our decision will be guided by the two goals of preserving the free status of all derivatives of our free software and of promoting the sharing and reuse of software generally.

NO WARRANTY

11. BECAUSE THE PROGRAM IS LICENSED FREE OF CHARGE, THERE IS NO WARRANTY FOR THE PROGRAM, TO THE EXTENT PERMITTED BY APPLICABLE LAW. EXCEPT WHEN OTHERWISE STATED IN WRITING THE COPYRIGHT HOLDERS AND/OR OTHER PARTIES PROVIDE THE PROGRAM "AS IS" WITHOUT WARRANTY OF ANY KIND, EITHER EXPRESSED OR IMPLIED, INCLUDING, BUT NOT LIMITED TO, THE IMPLIED WARRANTIES OF MERCHANTABILITY AND FITNESS FOR A PARTICULAR PURPOSE. THE ENTIRE RISK AS TO THE QUALITY AND PERFORMANCE OF THE PROGRAM IS WITH YOU. SHOULD THE PROGRAM PROVE DEFECTIVE, YOU ASSUME THE COST OF ALL NECESSARY SERVICING, REPAIR OR CORRECTION.

12. IN NO EVENT UNLESS REQUIRED BY APPLICABLE LAW OR AGREED TO IN WRITING WILL ANY COPYRIGHT HOLDER, OR ANY OTHER PARTY WHO MAY MODIFY AND/OR REDISTRIBUTE THE PROGRAM AS PERMITTED ABOVE, BE LIABLE TO YOU FOR DAMAGES, INCLUDING ANY GENERAL, SPECIAL, INCIDENTAL OR CONSEQUENTIAL DAMAGES ARISING OUT OF THE USE OR INABILITY TO USE THE PROGRAM (INCLUDING BUT NOT LIMITED TO LOSS OF DATA OR DATA BEING RENDERED INACCURATE OR LOSSES SUSTAINED BY YOU OR THIRD PARTIES OR A FAILURE OF THE PROGRAM TO OPERATE WITH ANY OTHER PROGRAMS), EVEN IF SUCH HOLDER OR OTHER PARTY HAS BEEN ADVISED OF THE POSSIBILITY OF SUCH DAMAGES.

END OF TERMS AND CONDITIONS

**S1. WinBUGS code to compare classifiers in the absence of a gold standard**

# WinBUGS code to compare classifiers in the absence of a gold standard

# Copyright (C) 2011 Monash University

# Written by Jonathan Keith

# This program is free software; you can redistribute it and/or

# modify it under the terms of the GNU General Public License

# as published by the Free Software Foundation; either version 2

# of the License, or (at your option) any later version.

# This program is distributed in the hope that it will be useful,

# but WITHOUT ANY WARRANTY; without even the implied warranty of

# MERCHANTABILITY or FITNESS FOR A PARTICULAR PURPOSE. See the

# GNU General Public License for more details.

# You should have received a copy of the GNU General Public License

# along with this program; if not, write to the Free Software

# Foundation, Inc., 51 Franklin Street, Fifth Floor, Boston, MA 02110-1301, USA.

# email: jonathan.keith@monash.edu

model

{

for(i in 1:N){ # N is the number of individuals

for(j in 1:K){ # K is the number of classifiers

# I

class[j,i] ~ dbern(params[j,diseasepl[i]])

}

# Same as disease but with offset 1, instead of 0

diseasepl[i] <- disease[i]+1

# Actual disease status ie true classification

disease[i] ~ dbern(pd)

}

# Distributions of classifier parameters

for(j in 1:K){

# Parameter for the distribution of the probability of getting a true positive

params[j,1] ~ dunif(0,params[j,2])

# Parameter for the distribution of the probability of getting a true negative

params[j,2] ~ dunif(params[j,1],1)

# Sensitivity

sens[j] <- params[j,2]

# Specificity

spec[j] <- 1-params[j,1]

}

# Distribution of prevalence

pd ~ dunif(0,1)

}

The code is also available from [this link](http://bioinformatics.monash.edu.au/downloads/).

**S2. Test data and initialisers for the model**

**S2.1. Swine flu**

**S2.1.1. Swine flu data**

list(N=48, K=2,

class=structure(.Data = c(1,1,1,1,1,1,1,1,1,1,1,1,1,1,1,1,0,0,0,0,0,0,0,0,0,0,0,0,0,0,0,0,0,0,0,0,0,0,0,0,0,0,0,0,0,0,0,0,

1,1,1,1,1,1,1,1,1,1,0,0,0,0,0,0,1,1,0,0,0,0,0,0,0,0,0,0,0,0,0,0,0,0,0,0,0,0,0,0,0,0,0,0,0,0,0,0),

.Dim = c(2,48)

)

)

**S2.1.2. Swine flu initialisers**

list(params=structure(.Data = c(0.5,0.5,0.5,0.5), .Dim=c(2,2)),

pd=0.5

)

**S2.2. Chloroplast subcellular localisation**

**S2.2.1. Chloroplast subcellular localisation data**

list(N=357, K=3,

class=structure(.Data = c(1,0,1,0,0,1,1,1,0,1,1,1,1,1,1,0,0,0,1,0,0,1,0,0,1,1,0,0,1,0,1,1,0,0,1,1,0,0,1,0,1,1,0,0,0,1,1,1,1,0,1,1,1,0,0,0,0,0,1,0,0,0,0,0,1,1,0,1,0,1,0,0,1,0,0,0,0,1,0,0,0,0,0,0,0,1,0,0,0,0,1,0,0,0,0,0,0,0,1,1,0,1,1,1,0,0,0,0,1,1,1,0,1,1,0,0,0,0,0,0,1,0,0,0,0,0,1,0,0,0,0,0,0,0,0,0,0,0,0,1,0,0,1,0,0,0,0,0,0,0,0,0,0,0,0,0,0,0,0,0,0,0,0,0,0,0,0,0,0,0,0,0,0,0,0,0,0,0,0,0,0,0,0,0,0,0,0,1,0,0,0,0,0,0,0,0,0,0,0,0,0,0,0,0,0,0,0,0,0,0,0,0,0,0,0,0,0,0,0,0,0,0,0,0,0,0,0,0,0,0,0,0,0,0,0,0,0,0,0,0,0,0,0,0,0,0,0,0,0,0,0,0,0,0,0,0,0,0,0,0,0,0,0,0,0,0,0,0,0,0,0,0,0,0,1,0,0,0,0,0,0,0,0,0,0,0,0,0,0,0,0,0,0,0,0,0,0,0,0,0,0,0,0,0,0,0,0,1,0,0,0,0,0,0,0,0,0,0,0,0,0,0,0,0,0,0,0,0,0,0,0,0,1,0,0,0,0,0,0,1,0,1,0,0,0,0,1,0,0,0,0,0,0,0,0,0,1,1,0,0,0,1,1,0,0,1,1,1,1,0,0,0,0,1,0,0,0,1,0,0,0,0,0,0,0,0,0,1,0,0,0,1,1,0,0,1,1,1,1,0,0,1,0,0,0,1,0,1,0,1,0,0,0,0,0,1,0,0,0,0,0,1,1,0,0,0,0,0,0,0,0,1,1,0,1,0,0,0,0,0,0,0,0,0,0,0,0,0,0,0,0,0,0,0,0,1,0,0,0,1,0,0,0,0,0,1,1,0,0,1,0,0,0,0,0,0,0,0,0,0,0,0,0,0,0,0,0,0,0,0,0,0,0,0,0,1,1,0,0,0,0,0,0,0,0,0,0,0,0,0,0,0,0,0,0,0,0,0,0,0,0,0,0,0,0,1,0,0,0,0,0,0,0,0,0,0,1,0,0,0,0,0,1,0,0,0,0,0,0,0,0,0,0,0,0,0,0,0,0,0,0,0,0,0,0,0,0,0,0,0,0,0,0,0,0,0,0,0,0,0,0,0,0,0,0,0,0,0,0,0,0,0,0,0,0,0,0,0,0,0,0,0,0,0,0,0,0,0,0,0,0,0,0,0,0,0,0,0,0,0,0,0,0,0,0,0,0,0,0,0,0,0,0,0,0,0,0,0,0,0,0,0,0,0,0,0,0,0,0,0,0,0,0,0,0,0,0,0,0,0,0,0,0,0,0,0,0,0,0,0,0,0,0,0,0,0,0,0,0,0,0,0,0,0,0,0,0,0,0,0,0,0,0,0,0,0,0,0,0,0,0,0,0,0,0,0,0,0,0,0,0,0,1,0,1,0,0,0,1,1,0,0,1,1,1,1,0,1,1,0,0,0,0,0,0,1,1,1,1,1,0,0,1,0,1,1,0,0,1,1,0,1,0,1,1,1,1,0,0,1,1,1,1,0,1,0,1,0,0,0,0,0,1,0,0,0,0,1,0,1,0,0,0,1,1,0,0,0,0,0,0,1,0,0,0,0,0,0,0,0,0,0,0,0,0,0,0,0,0,0,0,0,0,0,0,1,0,0,0,0,0,0,0,0,0,0,1,0,1,1,0,0,0,0,0,0,0,0,0,0,0,1,1,0,0,0,0,0,0,0,0,0,0,0,0,0,0,0,0,0,0,0,0,0,0,0,0,0,0,0,0,0,0,0,0,0,0,0,0,0,0,0,0,0,0,0,0,0,0,0,0,0,0,0,0,0,0,0,0,0,0,0,0,0,0,0,0,0,0,0,0,0,0,0,0,0,0,0,0,0,0,0,0,0,0,0,0,0,0,0,0,0,0,0,0,1,0,0,0,0,0,0,0,0,0,0,0,0,0,0,0,0,0,0,0,0,0,0,0,0,0,0,0,0,0,0,0,0,0,0,0,0,0,0,0,0,0,0,0,0,0,0,0,0,0,0,0,0,0,0,0,0,0,0,0,0,0,0,0,0,0,0,0,0,0,0,0,0,0,0,1,0,0,0,0,0,0,0,0,0,0,0,0,0,0,0,0,0,0,0,0,0,0,0,0,0,0,0,0,0,0,0,0,1,0,0,0,0,0,0,0,0,0,0,0,0,0,1,0,0,0,0,0,0,0,0,0,0,0,1,1), .Dim = c(3,357)

)

)

**S2.2.2. Chloroplast subcellular localisation initialisers**

list(params=structure(.Data = c(0.5,0.5,0.5,0.5,0.5,0.5), .Dim=c(3,2)),

pd=0.5

)

**S2.3. SNP**

**S2.3.1. SNP data**

This dataset is too large to display here. Please [click here](http://bioinformatics.monash.edu.au/downloads/) to obtain it.

**S2.3.2. SNP initialisers**

list(params=structure(.Data = c(0.5,0.5,0.5,0.5,0.5,0.5), .Dim=c(3,2)),

pd=0.5

)

**S3. Subcellular localisation results**

S3.1. Comparison of results for different subcellular locations

|  |  | **Chloroplast** | | | **Cytoplasm** | | | **Golgi Apparatus** | | | **Mitochondrion** | | | **Extracellular** | | | **Nuclear** | | | **Cell Membrane** | | |
| --- | --- | --- | --- | --- | --- | --- | --- | --- | --- | --- | --- | --- | --- | --- | --- | --- | --- | --- | --- | --- | --- | --- |
| Classifier |  | mean | sd | rank | mean | sd | rank | mean | sd | rank | mean | sd | rank | mean | sd | rank | mean | sd | rank | mean | sd | rank |
| AA | Actual Sens | 0.493 |  | 1 | 0.125 |  | 2 | 0.083 |  | 1 | 0.047 |  | 2 | 0.640 |  | 1 | 0.902 |  | 1 | 0.393 |  | 1 |
|  | Inferred | 0.785 | 0.090 | 1 | 0.411 | 0.218 | 2 | 0.561 | 0.260 | 1 | 0.388 | 0.279 | 2 | 0.933 | 0.062 | 1 | 0.990 | 0.010 | 1 | 0.599 | 0.198 | 1 |
|  | Actual Spec | 0.903 |  | 3 | 0.982 |  | 3 | 0.988 |  | 3 | 0.981 |  | 2 | 0.987 |  | 2 | 0.857 |  | 2 | 0.991 |  | 1^*^ |
|  | Inferred | 0.935 | 0.022 | 3 | 0.981 | 0.009 | 3 | 0.988 | 0.007 | 3 | 0.978 | 0.009 | 2 | 0.991 | 0.007 | 2 | 0.897 | 0.024 | 2 | 0.981 | 0.011 | 2^*^ |
| DP | Actual Sens | 0.343 |  | 3 | 0.083 |  | 3 | 0.083 |  | 1^*^ | 0.047 |  | 2^*^ | 0.240 |  | 3 | 0.827 |  | 2 | 0.143 |  | 3 |
|  | Inferred | 0.553 | 0.094 | 3 | 0.258 | 0.193 | 3 | 0.462 | 0.268 | 2^*^ | 0.313 | 0.263 | 3^*^ | 0.402 | 0.083 | 3 | 0.886 | 0.035 | 2 | 0.373 | 0.179 | 3 |
|  | Actual Spec | 0.952 |  | 1 | 0.997 |  | 1 | 0.997 |  | 1 | 0.987 |  | 1 | 0.993 |  | 1 | 0.915 |  | 1 | 0.991 |  | 1 |
|  | Inferred | 0.971 | 0.013 | 1 | 0.992 | 0.005 | 1 | 0.995 | 0.004 | 1 | 0.982 | 0.008 | 1 | 0.997 | 0.003 | 1 | 0.938 | 0.017 | 1 | 0.990 | 0.006 | 1 |
| NCC | Actual Sens | 0.493 |  | 1^*^ | 0.208 |  | 1 | 0.000 |  | 3 | 0.256 |  | 1 | 0.560 |  | 2 | 0.782 |  | 3 | 0.179 |  | 2 |
|  | Inferred | 0.687 | 0.099 | 2^*^ | 0.561 | 0.242 | 1 | 0.271 | 0.219 | 3 | 0.455 | 0.265 | 1 | 0.722 | 0.080 | 2 | 0.873 | 0.033 | 3 | 0.469 | 0.177 | 2 |
|  | Actual Spec | 0.948 |  | 2 | 0.991 |  | 2 | 0.997 |  | 1^*^ | 0.959 |  | 3 | 0.889 |  | 3 | 0.853 |  | 3 | 0.982 |  | 3 |
|  | Inferred | 0.959 | 0.016 | 2 | 0.987 | 0.008 | 2 | 0.992 | 0.005 | 2^*^ | 0.933 | 0.017 | 3 | 0.960 | 0.012 | 3 | 0.865 | 0.024 | 3 | 0.970 | 0.012 | 3 |

* These differences between the inferred and the actual rankings are in all cases due to the actual rankings of two classifiers being tied.

S3.2. Table of chloroplast localisation statistics

| **node** | **mean** | **sd** | **MC error** | **2.5%** | **median** | **97.5%** | **start** | **sample** |
| --- | --- | --- | --- | --- | --- | --- | --- | --- |
| params[1,1] | 0.06521 | 0.0216 | 8.55E-04 | 0.02025 | 0.06545 | 0.1076 | 5000 | 5001 |
| params[1,2] | 0.7851 | 0.08967 | 0.003347 | 0.5944 | 0.7908 | 0.9422 | 5000 | 5001 |
| params[2,1] | 0.02929 | 0.0126 | 3.73E-04 | 0.006747 | 0.02847 | 0.05594 | 5000 | 5001 |
| params[2,2] | 0.5525 | 0.09393 | 0.003281 | 0.3739 | 0.5528 | 0.7366 | 5000 | 5001 |
| params[3,1] | 0.04102 | 0.01601 | 5.54E-04 | 0.0117 | 0.04042 | 0.07402 | 5000 | 5001 |
| params[3,2] | 0.6872 | 0.09904 | 0.003874 | 0.4872 | 0.6909 | 0.8688 | 5000 | 5001 |
| pd | 0.1511 | 0.03384 | 0.001548 | 0.09623 | 0.1469 | 0.2301 | 5000 | 5001 |
| sens[1] | 0.7851 | 0.08967 | 0.003347 | 0.5944 | 0.7908 | 0.9422 | 5000 | 5001 |
| sens[2] | 0.5525 | 0.09393 | 0.003281 | 0.3739 | 0.5528 | 0.7366 | 5000 | 5001 |
| sens[3] | 0.6872 | 0.09904 | 0.003874 | 0.4872 | 0.6909 | 0.8688 | 5000 | 5001 |
| spec[1] | 0.9348 | 0.0216 | 8.55E-04 | 0.8924 | 0.9346 | 0.9798 | 5000 | 5001 |
| spec[2] | 0.9707 | 0.0126 | 3.73E-04 | 0.9441 | 0.9715 | 0.9933 | 5000 | 5001 |
| spec[3] | 0.959 | 0.01601 | 5.54E-04 | 0.926 | 0.9596 | 0.9883 | 5000 | 5001 |

params[1,1] : probability of a true positive for AA

params[1,2] : probability of a false positive for AA

params[2,1] : probability of a true positive for DP

params[2,2] : probability of a false positive for DP

params[3,1] : probability of a true positive for NCC

params[3,2] : probability of a false positive for NCC

pd : probability that a given protein is localised to the chloroplast region

sens[1] : sensitivity of AA

sens[2] : sensitivity of DP

sens[3] : sensitivity of NCC

spec[1] : specificity of AA

spec[2] : specificity of DP

spec[3] : specificity of NCC

**S3.3. Time-series plots for chloroplast localisation**

The model and chloroplast data were loaded into WinBUGS. The model and data were then compiled and the model variables were initialised, before running the simulation. The following plots show the evolution of selected model parameters over 10000 iterations.

1. Probability of belonging to chloroplast region (phi). N.B. In the WinBUGS model this quantity is denoted ‘pd’.

*
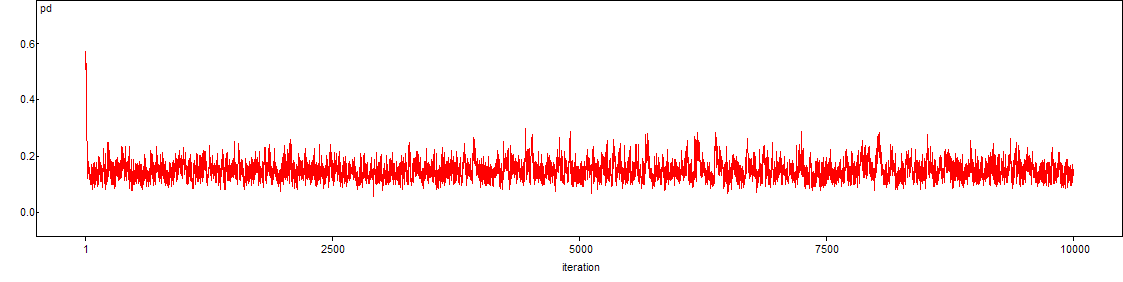
*

1. Sensitivity of the amino acid composition-based SVM classifier (AA).

*
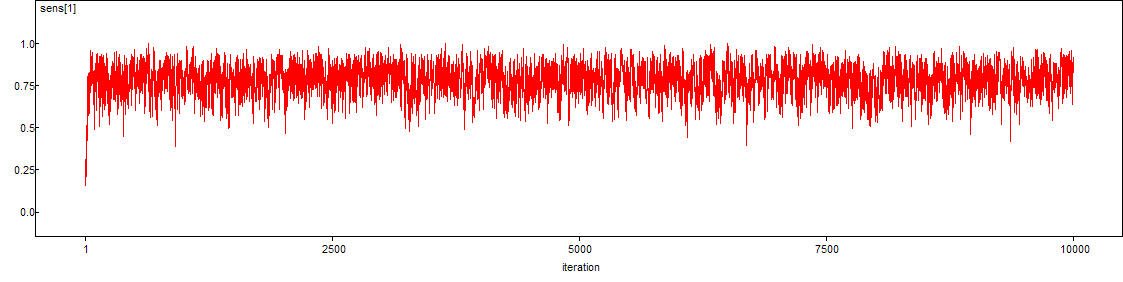
*

1. Sensitivity of the dipeptide composition-based classifier (DP).

*
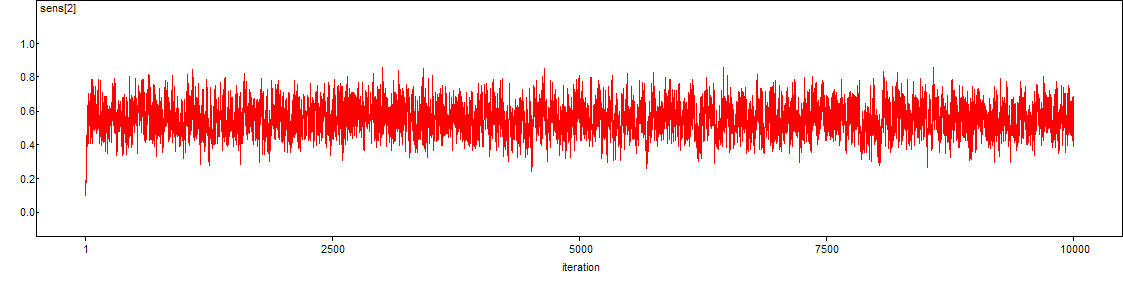
*

1. Sensitivity of the N-center-C terminal region-based classifier (NCC).

*
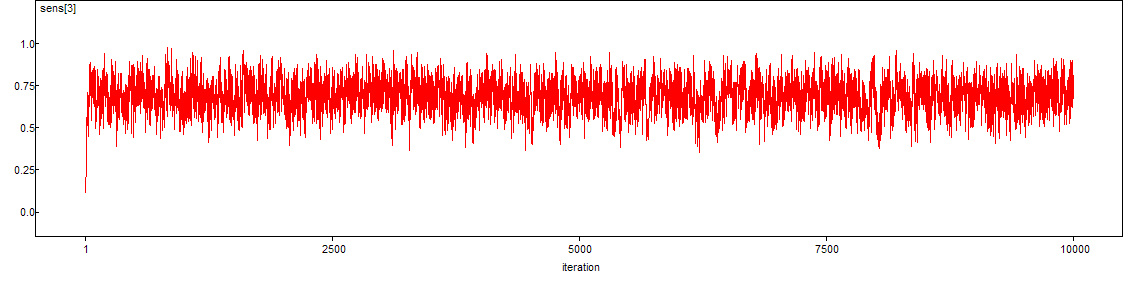
*

1. Specificity of the amino acid composition-based SVM classifier (AA).

*
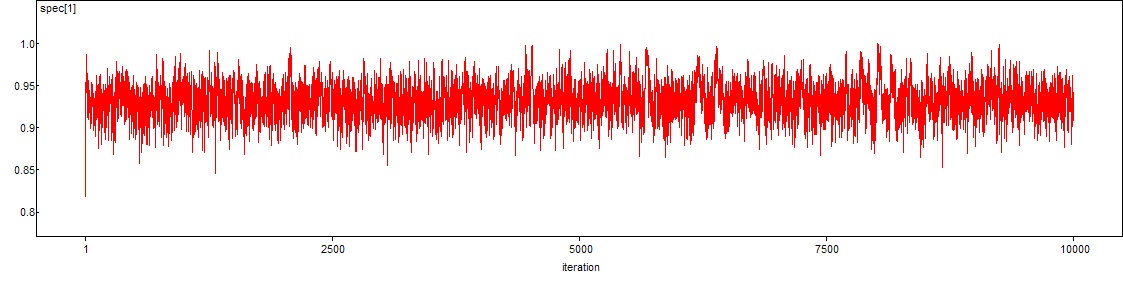
*

1. Specificity of the dipeptide composition-based classifier (DP).

*
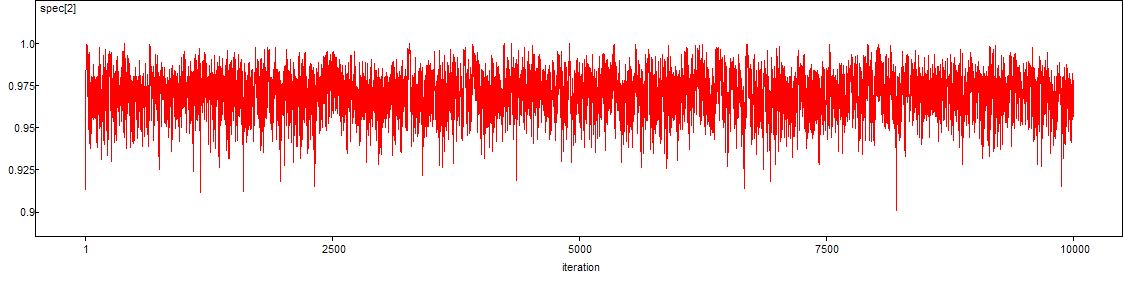
*

1. Specificity of the N-Center-C terminal region-based classifier (NCC).

*
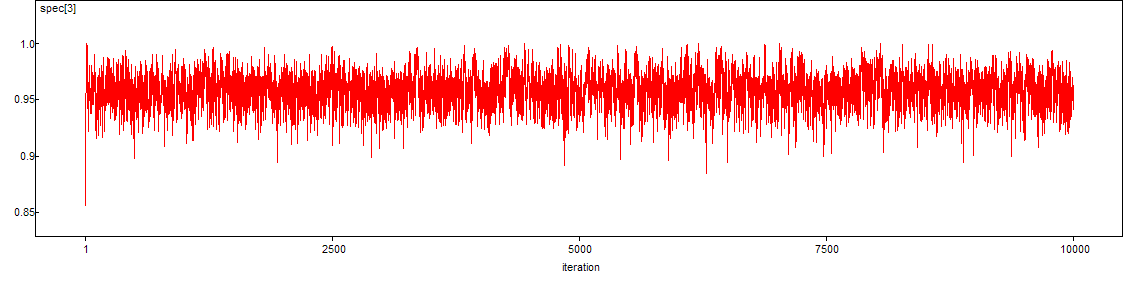
*

**S4. Swine flu results**

S4.1. Swine flu statistics

| **node** | **mean** | **sd** | **MC error** | **2.5%** | **median** | **97.5%** | **start** | **sample** |
| --- | --- | --- | --- | --- | --- | --- | --- | --- |
| params[1,1] | 0.1215 | 0.07741 | 0.002625 | 0.006384 | 0.1146 | 0.2931 | 5000 | 5001 |
| params[1,2] | 0.8409 | 0.1163 | 0.003417 | 0.5692 | 0.863 | 0.9931 | 5000 | 5001 |
| params[2,1] | 0.06761 | 0.05112 | 0.001314 | 0.003098 | 0.05728 | 0.1914 | 5000 | 5001 |
| params[2,2] | 0.7297 | 0.1568 | 0.00606 | 0.4158 | 0.7337 | 0.9833 | 5000 | 5001 |
| pd | 0.3042 | 0.09079 | 0.003443 | 0.1447 | 0.298 | 0.4944 | 5000 | 5001 |
| sens[1] | 0.8409 | 0.1163 | 0.003417 | 0.5692 | 0.863 | 0.9931 | 5000 | 5001 |
| sens[2] | 0.7297 | 0.1568 | 0.00606 | 0.4158 | 0.7337 | 0.9833 | 5000 | 5001 |
| spec[1] | 0.8785 | 0.07741 | 0.002625 | 0.7069 | 0.8854 | 0.9936 | 5000 | 5001 |
| spec[2] | 0.9324 | 0.05112 | 0.001314 | 0.8086 | 0.9427 | 0.9969 | 5000 | 5001 |

params[1,1] : probability of a true positive for NPA

params[1,2] : probability of a false positive for NPA

params[2,1] : probability of a true positive for NS

params[2,2] : probability of a false positive for NS

pd : probability of a selected individual in the group having swine flu

sens[1] : sensitivity of NPA

sens[2] : sensitivity of NS

spec[1] : specificity of NPA

spec[2] : specificity of NS

**S4.2. Time-series plots of swine flu results**

The model and swine flu data were loaded into WinBUGS. The model and data were then compiled and the model variables were initialised, before running the simulation. The following plots show the evolution of selected model parameters over 10000 iterations.

1. Estimated prevalence of disease (phi). N.B. In the WinBUGS model this quantity is denoted ‘pd’.


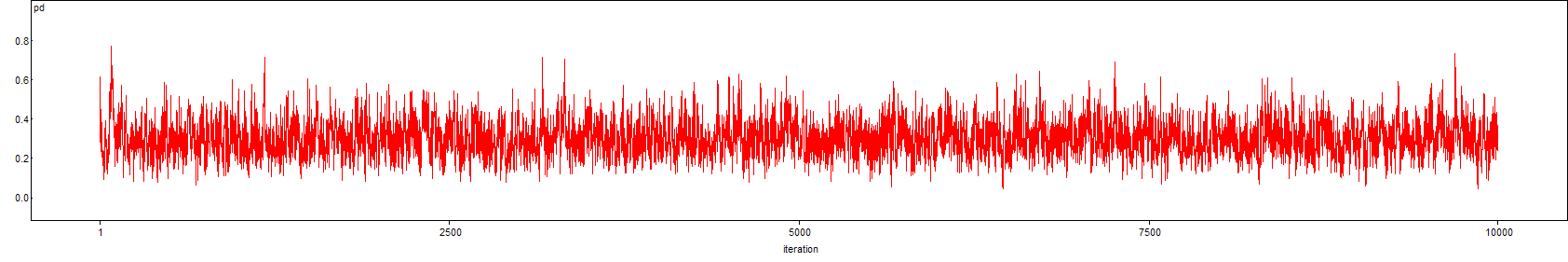


1. Sensitivity of the NPA classifier.


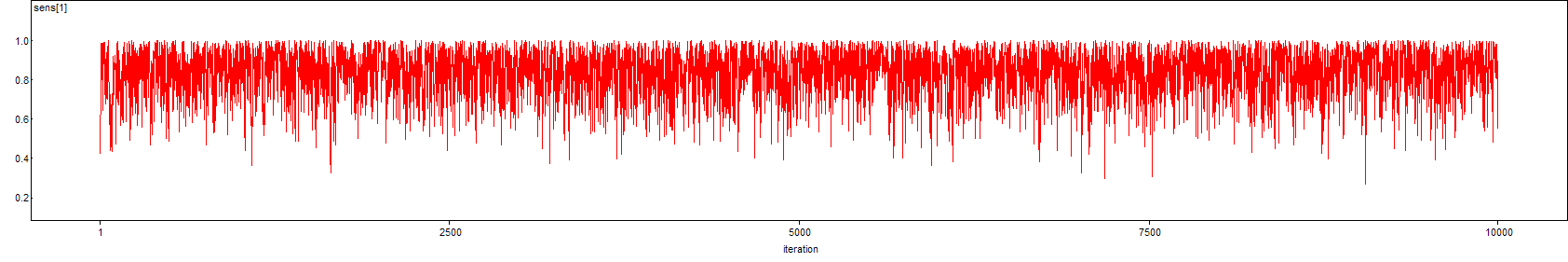


1. Sensitivity of the NS classifier.

*
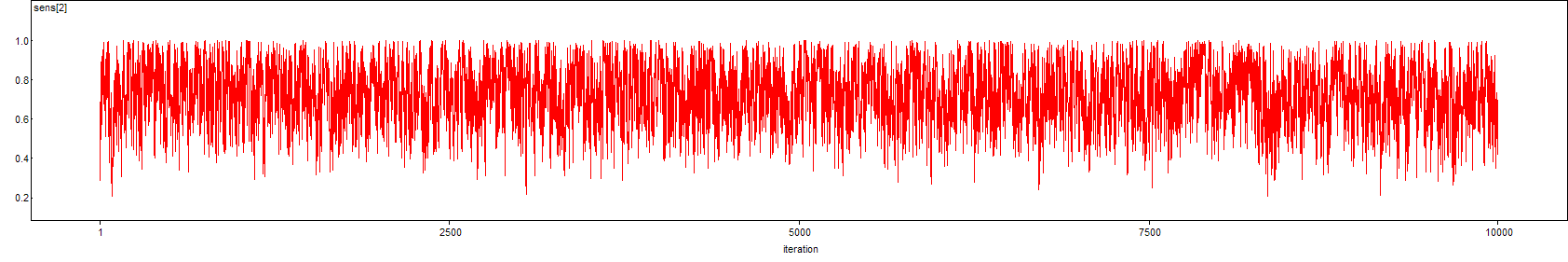
*

1. Specificity of the NPA classifier.

*
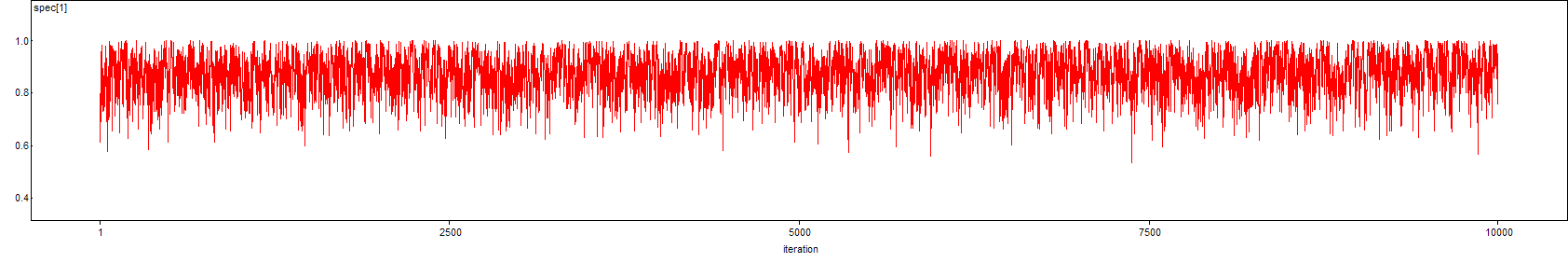
*

1. Specificity of the NS classifier.

*
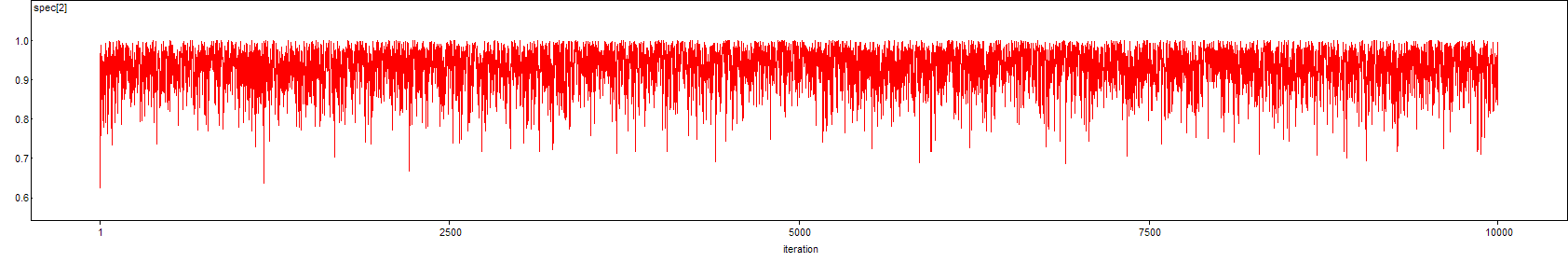
*

**S5. SNP results**

**S5.1. Table of SNP statistics**

~1000 positives per classifier

| **node** | **mean** | **sd** | **MC error** | **2.50%** | **median** | **97.50%** | **start** | **sample** |
| --- | --- | --- | --- | --- | --- | --- | --- | --- |
| pd | 0.001535 | 1.469E-4 | 1.099E-5 | 0.001283 | 0.001525 | 0.001857 | 5001 | 5000 |
| sens[1] | 0.8356 | 0.04007 | 0.002693 | 0.7506 | 0.8372 | 0.9069 | 5001 | 5000 |
| sens[2] | 0.5693 | 0.04266 | 0.0029 | 0.4899 | 0.5682 | 0.6545 | 5001 | 5000 |
| sens[3] | 0.1691 | 0.01819 | 9.257E-4 | 0.1359 | 0.1685 | 0.206 | 5001 | 5000 |
| spec[1] | 0.9994 | 9.337E-5 | 6.612E-6 | 0.9992 | 0.9994 | 0.9996 | 5001 | 5000 |
| spec[2] | 0.999 | 5.826E-5 | 2.765E-6 | 0.9989 | 0.999 | 0.9991 | 5001 | 5000 |
| spec[3] | 0.9978 | 6.417E-5 | 1.003E-6 | 0.9977 | 0.9978 | 0.998 | 5001 | 5000 |

~5000 positives per classifier

| **node** | **mean** | **sd** | **MC error** | **2.50%** | **median** | **97.50%** | **start** | **sample** |
| --- | --- | --- | --- | --- | --- | --- | --- | --- |
| pd | 0.009506 | 5.01E-4 | 4.969E-5 | 0.008601 | 0.009488 | 0.01055 | 5001 | 5000 |
| sens[1] | 0.8283 | 0.02172 | 0.001863 | 0.7841 | 0.8281 | 0.87 | 5001 | 5000 |
| sens[2] | 0.5468 | 0.0214 | 0.002002 | 0.5036 | 0.5467 | 0.5875 | 5001 | 5000 |
| sens[3] | 0.1532 | 0.007755 | 5.782E-4 | 0.1383 | 0.153 | 0.1687 | 5001 | 5000 |
| spec[1] | 0.9986 | 2.991E-4 | 2.918E-5 | 0.9981 | 0.9986 | 0.9992 | 5001 | 5000 |
| spec[2] | 0.9952 | 1.612E-4 | 1.128E-5 | 0.9949 | 0.9952 | 0.9956 | 5001 | 5000 |
| spec[3] | 0.9921 | 1.252E-4 | 2.627E-6 | 0.9919 | 0.9921 | 0.9923 | 5001 | 5000 |

pd : probability that any given gene is associated with age-related macular degeneration

sens[1] : sensitivity of PLINK

sens[2] : sensitivity of the gene-based method

sens[3] : sensitivity of the interaction method

spec[1] : specificity of PLINK

spec[2] : specificity of the gene-based method

spec[3] : specificity of the interaction method

S5.2. Density plots of SNP results with ~1000 positives

| 1. *Sensitivity of the PLINK classifier*   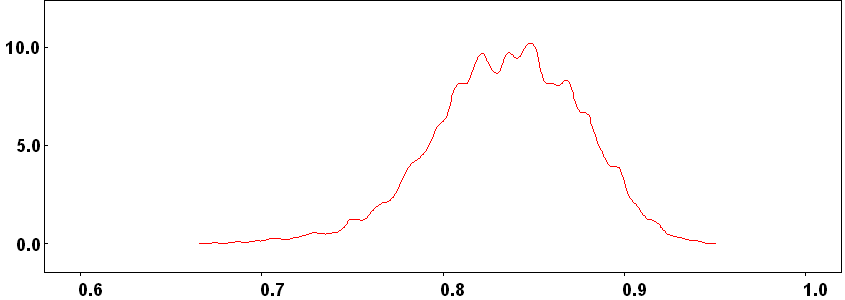 | 1. *Specificity of the PLINK classifier*   *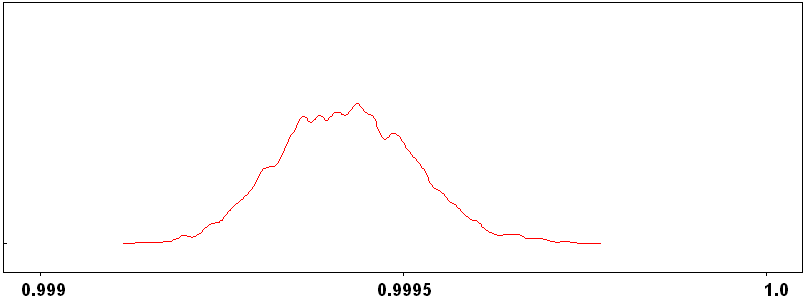* |
| --- | --- |
| 1. *Sensitivity of the gene-based classifier*   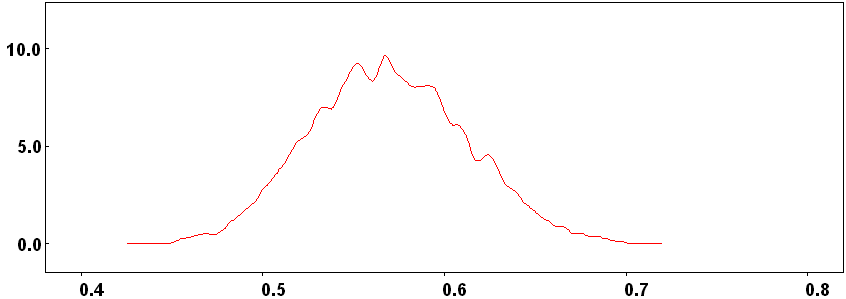 | 1. *Specificity of the gene-based classifier*   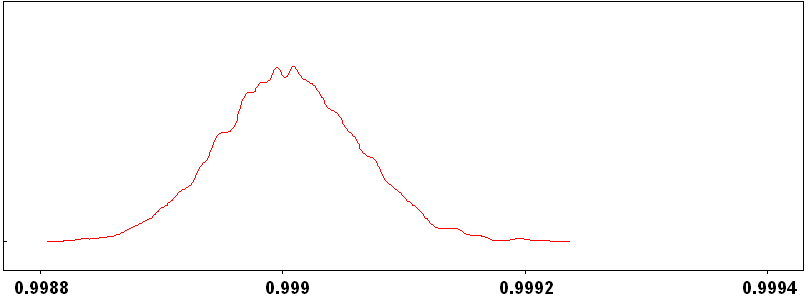 |
| 1. *Sensitivity of the interaction count-based classifier*   *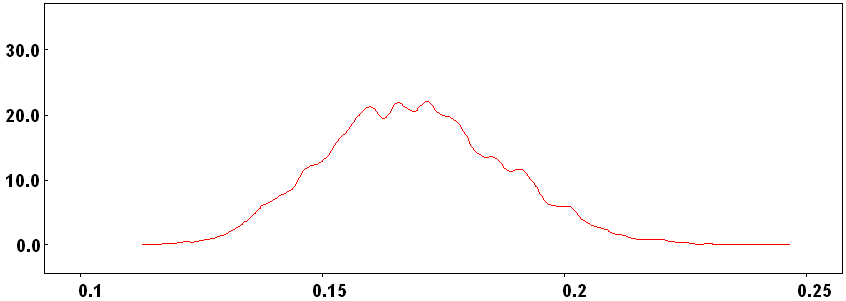* | 1. *Specificity of the interaction count-based classifier*   *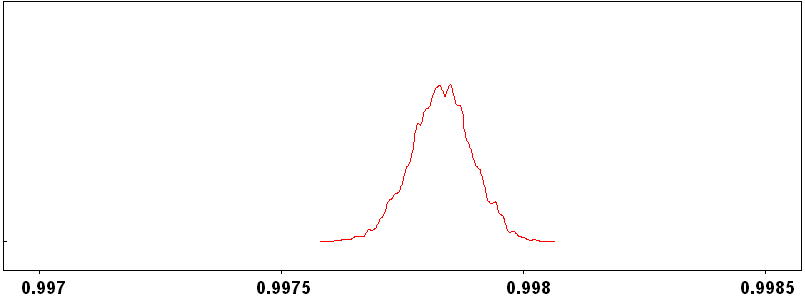* |
| 1. *Probability that a given gene is associated with age-related macular degeneration*   *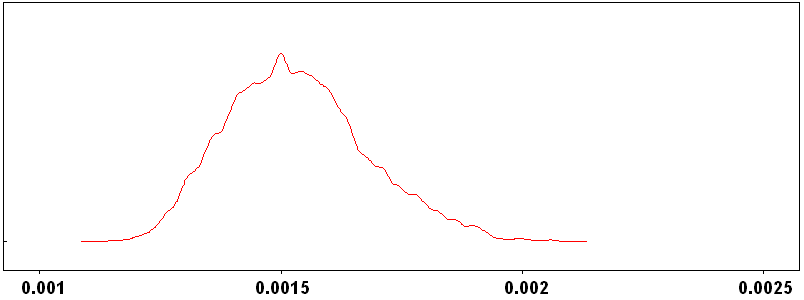* | |

S5.3. Density plots of SNP results with ~5000 positives

| 1. *Sensitivity of the PLINK classifier*   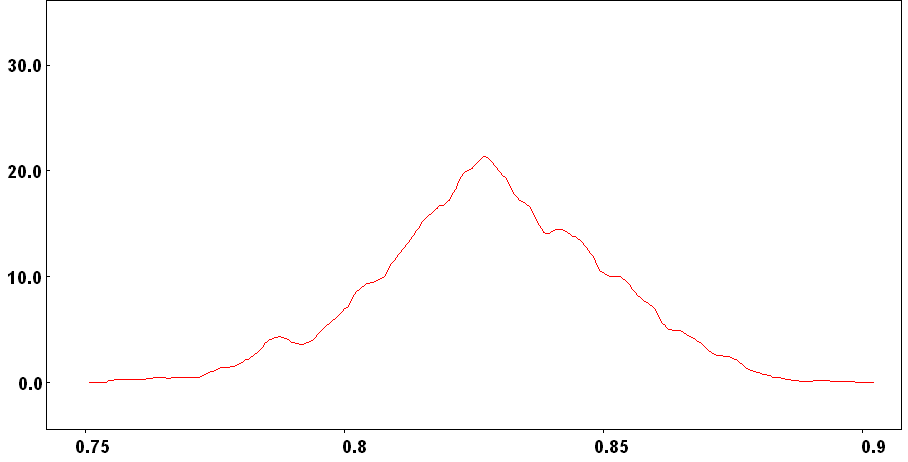 | 1. *Specificity of the PLINK classifier*   *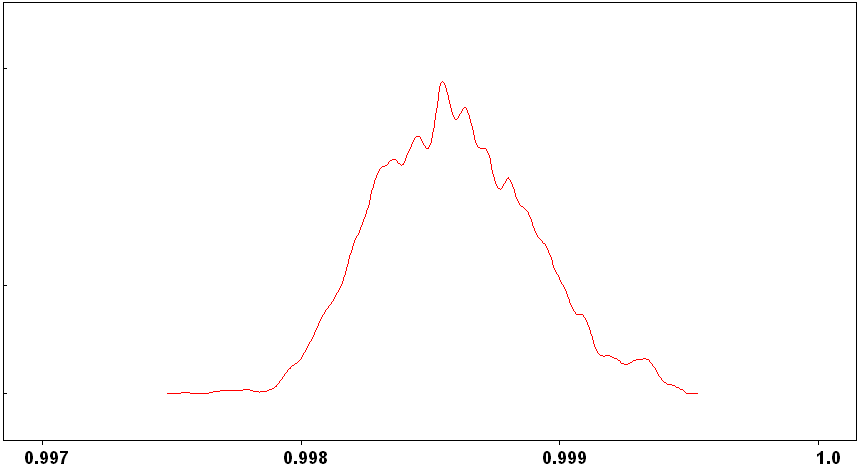* |
| --- | --- |
| 1. *Sensitivity of the gene-based classifier*   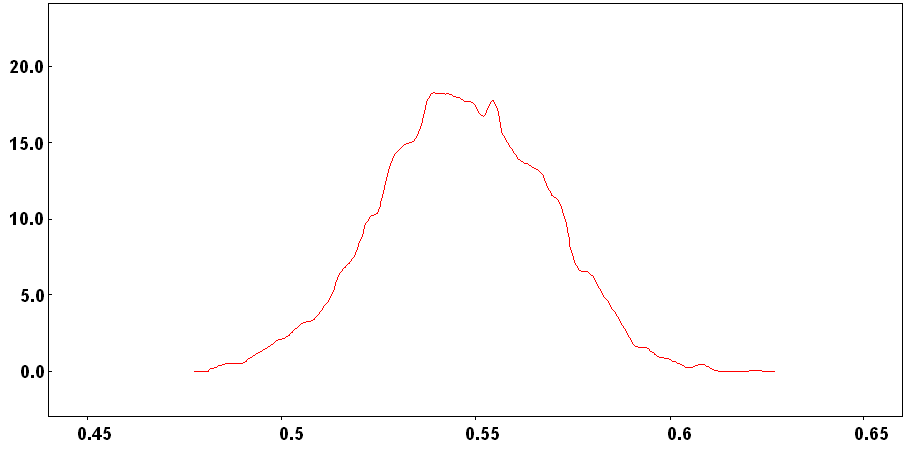 | 1. *Specificity of the gene-based classifier*   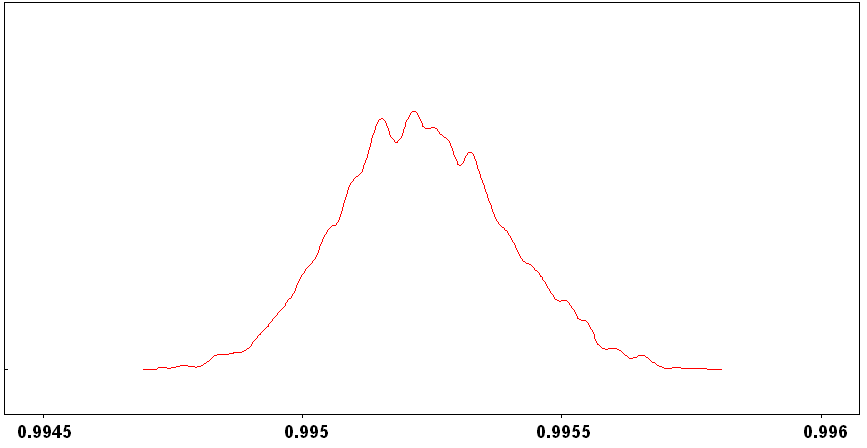 |
| 1. *Sensitivity of the interaction count-based classifier*   *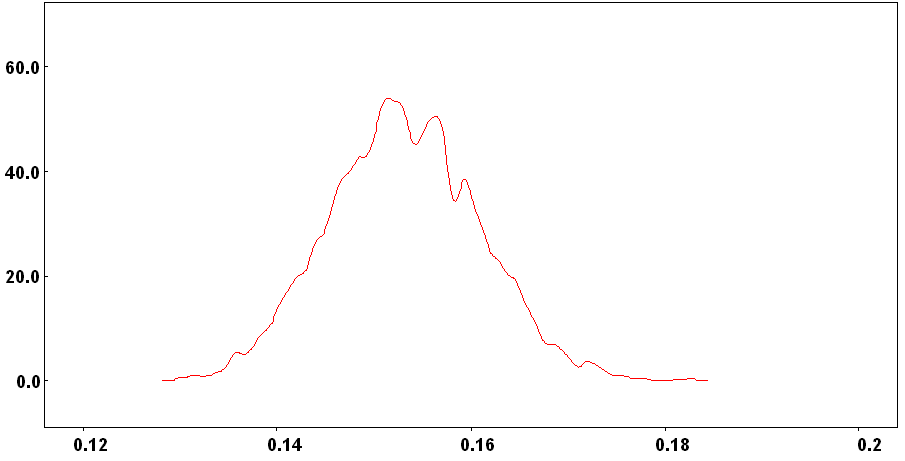* | 1. *Specificity of the interaction count-based classifier*   *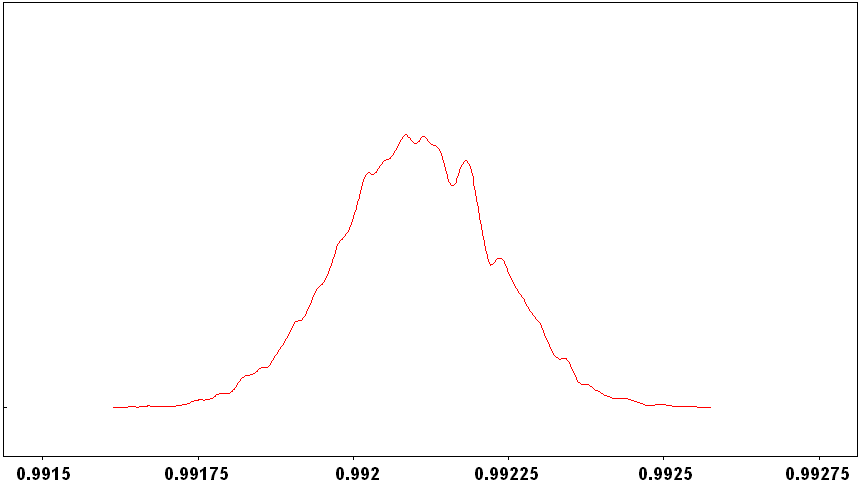* |
| 1. *Probability that a given gene is associated with age-related macular degeneration*   *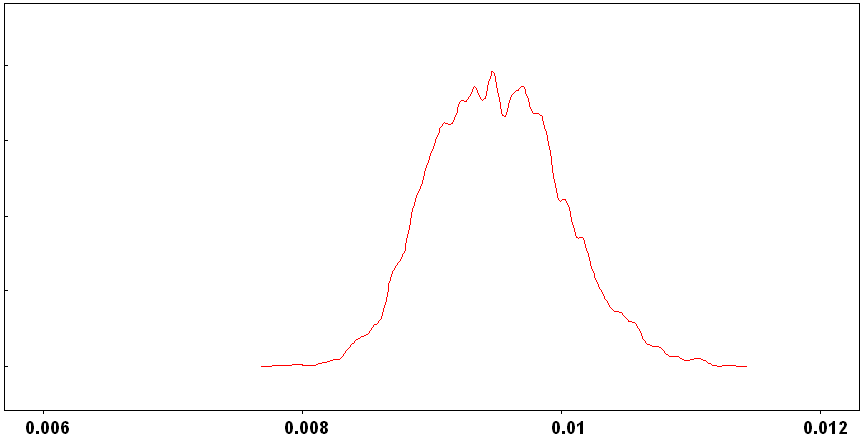* | |

**S6. R code to compare combinations of classifiers**

**S6.1. Identifying the most frequently best combination**

The following code was run to produce the swine flu combination comparison with 2000 iterations. The default behaviour of discarding the first half of the iterations was employed, so only the last 1000 iterations were passed to R. This code performs analysis using the last 500 iterations.

# R code to evaluate classifier combinations

# Copyright (C) 2011 Monash University

# Written by Christian Davey

# This program is free software; you can redistribute it and/or

# modify it under the terms of the GNU General Public License

# as published by the Free Software Foundation; either version 2

# of the License, or (at your option) any later version.

# This program is distributed in the hope that it will be useful,

# but WITHOUT ANY WARRANTY; without even the implied warranty of

# MERCHANTABILITY or FITNESS FOR A PARTICULAR PURPOSE. See the

# GNU General Public License for more details.

# You should have received a copy of the GNU General Public License

# along with this program; if not, write to the Free Software

# Foundation, Inc., 51 Franklin Street, Fifth Floor, Boston, MA 02110-1301, USA.

# email: jonathan.keith@monash.edu

####################################################################################

## Run Compare Classifiers WinBUGS Model from R and Evaluate Classifier Combinations

##

## Written by Christian Davey

## November 16, 2011

##

## For use with R (with the package R2WinBUGS) and WinBUGS14.

####################################################################################

# First run the model in WinBUGS14 and dump the output into R

# Load the R2WinBUGS package

library("R2WinBUGS")

# Load the data

data.file <- "snp_data_5000.txt"

# Initialise model variables

inits.file <- "snp_inits_5000.txt"

# Load the model

model <- "compare_classifiers_model.txt"

# Record the parameters to save

parameters.to.save=c("sens","spec")

# Record the number of chains

n.chains=1

# Record the number of iterations

n.iter=2000

# Run the model

classifiers.sim <- bugs(data=data.file,inits=inits.file,model.file=model,parameters.to.save,n.chains,n.iter,bugs.directory="C:/Program Files/WinBUGS14/",debug=TRUE)

# Load the WinBUGS output into the R workspace

attach.bugs(classifiers.sim)

# Now evaluate the classifiers

# Creaete an array to record the combinations that make up each classifier. Will be a 2^N*N matrix of 1s and 0s, where a 0 in the {i,j}th position indicates to intersect with classifier j in combination i.

comboarray <- NULL

N <- ncol(sens)

for(i in 1:2^N-1){

newrow <- NULL

for(j in 1:N){

newrow <- cbind(newrow,(1-(-1)^floor(i/(2^(j-1))))/2)

}

comboarray <- rbind(comboarray,newrow)

}

# Calculate the sensitivity and specificity of 'classifiers' created by intersecting actual classifiers with each other

# Initialise sensitivity and specificity vectors

sensitivity <- array(1,dim=c(2^N,500))

specificity <- array(0,dim=c(2^N,500))

for(i in 1:2^N){

for(j in 1:N){

for(k in 501:1000){

sensitivity[i,(k-500)] <- sensitivity[i,(k-500)]*((1-sens[k,j])^comboarray[i,j])*(sens[k,j]^(1-comboarray[i,j]))

}

}

for(j in 1:N){

for(k in 501:1000){

specificity[i,(k-500)] <- specificity[i,(k-500)]+((1-spec[k,j])^comboarray[i,j])*(spec[k,j]^(1-comboarray[i,j]))-specificity[i,(k-500)]*((1-spec[k,j])^comboarray[i,j])*(spec[k,j]^(1-comboarray[i,j]))

}

}

}

# Calculate sensitivities and specificities of unions of the intersections of classifiers

# Create another comboarray, where a 0 in position [i,j] indicates not to include intersection j in union i.

comboarray2 <- NULL

for(i in 1:2^(2^N)-1){

newrow <- NULL

for(j in 1:2^N){

newrow <- cbind(newrow,(1-(-1)^floor(i/(2^(j-1))))/2)

}

comboarray2 <- rbind(comboarray2,newrow)

}

# Initialise arrays for the sensitivity and specificity of these unions of intersections of classifiers

sensitivity2 <- array(0,dim=c(2^(2^N),500))

specificity2 <- array(1,dim=c(2^(2^N),500))

# Calculate the sensitivities and specificities

for(k in 1:500){

for(i in 1:(2^(2^N))){

for(j in 1:(2^N)){

sensitivity2[i,k] <- sensitivity2[i,k]+sensitivity[j,k]*comboarray2[i,j]

}

for(j in 1:2^N){

specificity2[i,k] <- specificity2[i,k]+(specificity[j,k]-1)*comboarray2[i,j]

}

}

}

# Pick out the best combination based on each of four different ranking methods

# First method: Product of Sensitivity and Specificity

bestcombo <- array(0,dim=c(1,500))

bestrank1 <- array(0,dim=c(1,500))

for(i in 1:(2^(2^N))){

for(k in 1:500){

if(sensitivity2[i,k]*specificity2[i,k] > bestcombo[k]){

bestcombo[k] <- sensitivity2[i,k]*specificity2[i,k]

bestrank1[k] <- i

}

}

}

# Second method: Sum of Squares of Sensitivity and Specificity

bestcombo <- array(0,dim=c(1,500))

bestrank2 <- array(0,dim=c(1,500))

for(i in 1:(2^(2^N))){

for(k in 1:500){

if(sensitivity2[i,k]^2+specificity2[i,k]^2 > bestcombo[k]){

bestcombo[k] <- (sensitivity2[i,k]^2+specificity2[i,k]^2)

bestrank2[k] <- i

}

}

}

# Third method: Sum of Absolute Values of Sensitivity and Specificity

bestcombo <- array(0,dim=c(1,500))

bestrank3 <- array(0,dim=c(1,500))

for(i in 1:(2^(2^N))){

for(k in 1:500){

if(abs(sensitivity2[i,k])+abs(specificity2[i,k]) > bestcombo[k]){

bestcombo[k] <- (abs(sensitivity2[i,k])+abs(specificity2[i,k]))

bestrank3[k] <- i

}

}

}

# Fourth method: Minimum of Sensitivity and Specificity

bestcombo <- array(0,dim=c(1,500))

bestrank4 <- array(0,dim=c(1,500))

for(i in 1:(2^(2^N))){

for(k in 1:500){

if(min(sensitivity2[i,k],specificity2[i,k]) > bestcombo[k]){

bestcombo[k] <- min(sensitivity2[i,k],specificity2[i,k])

bestrank4[k] <- i

}

}

}

# Record the combinations (this is how you calculate mode in R)

best1 <- as.numeric(names(which.max(table(bestrank1))))

best2 <- as.numeric(names(which.max(table(bestrank2))))

best3 <- as.numeric(names(which.max(table(bestrank3))))

best4 <- as.numeric(names(which.max(table(bestrank4))))

comboname1 <- ""

for(i in 1:ncol(comboarray2)){

comboname1 <- paste(comboname1,comboarray2[best1,i],sep="")

}

comboname2 <- ""

for(i in 1:ncol(comboarray2)){

comboname2 <- paste(comboname2,comboarray2[best2,i],sep="")

}

comboname3 <- ""

for(i in 1:ncol(comboarray2)){

comboname3 <- paste(comboname3,comboarray2[best3,i],sep="")

}

comboname4 <- ""

for(i in 1:ncol(comboarray2)){

comboname4 <- paste(comboname4,comboarray2[best4,i],sep="")

}

# Determine the probability that the mode is correct for each method

chancecorrect1 <- as.numeric(table(bestrank1)[names(which.max(table(best1)))])/500

chancecorrect2 <- as.numeric(table(bestrank2)[names(which.max(table(best2)))])/500

chancecorrect3 <- as.numeric(table(bestrank3)[names(which.max(table(best3)))])/500

chancecorrect4 <- as.numeric(table(bestrank4)[names(which.max(table(best4)))])/500

# Print the results

print(paste("According to Method 1 there is a probability of ",chancecorrect1, " that combination ", best1, " (", comboname1, ")", " is the best combination, with median sensitivity ", median(sensitivity2[best1,]), " and median specificity ", median(specificity2[best1,]), ".", sep=""))

print("This combination is a union of the following intersections (given as a binary code.)")

for(i in 1:2^N){

if(comboarray2[best1,i] == 1){

print(comboarray[i,])

}

}

print(paste("According to Method 1 there is a probability of ",chancecorrect2, " that combination ", best2, " (", comboname2, ")", " is the best combination, with median sensitivity ", median(sensitivity2[best2,]), " and median specificity ", median(specificity2[best2,]), ".", sep=""))

print("This combination is a union of the following intersections (given as a binary code.)")

for(i in 1:2^N){

if(comboarray2[best2,i] == 1){

print(comboarray[i,])

}

}

print(paste("According to Method 1 there is a probability of ",chancecorrect3, " that combination ", best3, " (", comboname3, ")", " is the best combination, with median sensitivity ", median(sensitivity2[best3,]), " and median specificity ", median(specificity2[best3,]), ".", sep=""))

print("This combination is a union of the following intersections (given as a binary code.)")

for(i in 1:2^N){

if(comboarray2[best3,i] == 1){

print(comboarray[i,])

}

}

print(paste("According to Method 1 there is a probability of ",chancecorrect4, " that combination ", best4, " (", comboname4, ")", " is the best combination, with median sensitivity ", median(sensitivity2[best4,]), " and median specificity ", median(specificity2[best4,]), ".", sep=""))

print("This combination is a union of the following intersections (given as a binary code.)")

for(i in 1:2^N){

if(comboarray2[best4,i] == 1){

print(comboarray[i,])

}

}

The code is also available from [this link](http://bioinformatics.monash.edu.au/downloads/).

**S6.2. Summary statistics for the combination ranking methods**

The following code was used to generate the tables of summary statistics for the four ranking methods shown in Sections S7.1.3, S7.2.3 and S7.3 below.

# R script to produce a table of the ranking values of the combinations of classifiers

# Change the separator values to whatever the separators are in the input files

sensitivity <- read.table(file="sensitivity2.txt",sep=",");

specificity <- read.table(file="specificity2.txt",sep=",");

N <- dim(sensitivity)[1];

# Calculate the ranking values for each combination

rank <- array(dim=c(N,8))

# First method: Product of Sensitivity and Specificity

for(i in 1:N){

rank[i,1] <- mean(as.numeric(sensitivity[i,]*specificity[i,]));

rank[i,2] <- sd(as.numeric(sensitivity[i,]*specificity[i,]));

}

print("Ranked by first method.");

# Second method: Sum of Squares of Sensitivity and Specificity

for(i in 1:N){

rank[i,3] <- mean(as.numeric(sensitivity[i,]^2+specificity[i,]^2));

rank[i,4] <- sd(as.numeric(sensitivity[i,]^2+specificity[i,]^2));

}

print("Ranked by second method.");

# Third method: Sum of Absolute Values of Sensitivity and Specificity

for(i in 1:N){

rank[i,5] <- mean(as.numeric(abs(sensitivity[i,])+abs(specificity[i,])));

rank[i,6] <- sd(as.numeric(abs(sensitivity[i,])+abs(specificity[i,])));

}

print("Ranked by third method.");

# Fourth method: Minimum of Sensitivity and Specificity

for(i in 1:N){

method4 <- array(dim=c(1,500));

for(j in 1:500){

method4[1,j] <- min(sensitivity[i,j],specificity[i,j]);

}

rank[i,7] <- mean(as.numeric(method4));

rank[i,8] <- sd(as.numeric(method4));

}

print("Ranked by fourth method.");

write.table(rank,file="rank.csv",sep=",",row.names=FALSE,col.names=FALSE);

The code is also available from [this link](http://bioinformatics.monash.edu.au/downloads/).

**S7. Density plots and summary statistics of selected combinations of classifiers**

**S7.1. Subcellular localisation (chloroplast)**

S7.1.1. Sensitivities of combinations

| 1. *Combination 2:* C_1_ΛC_2_ΛC_3_   *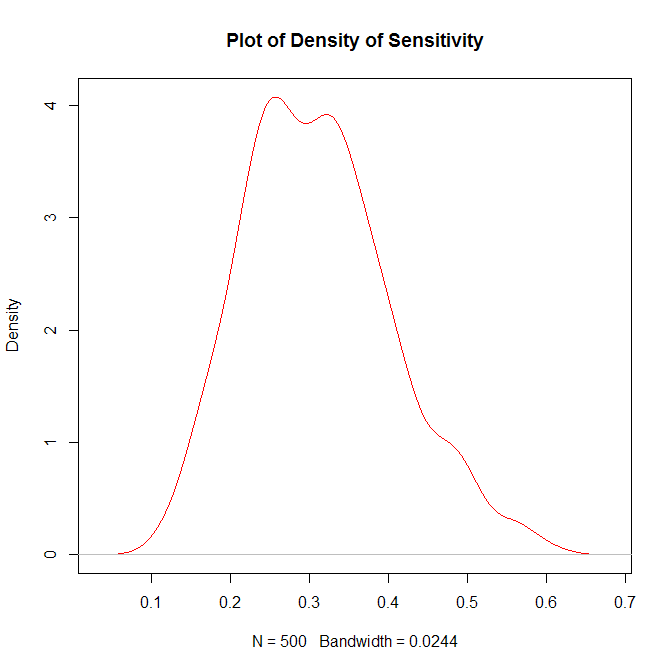* | 1. *Combination 4:* C_2_ΛC_3_=(C_1_ΛC_2_ΛC_3_)V(¬C_1_ΛC_2_ΛC_3_)   *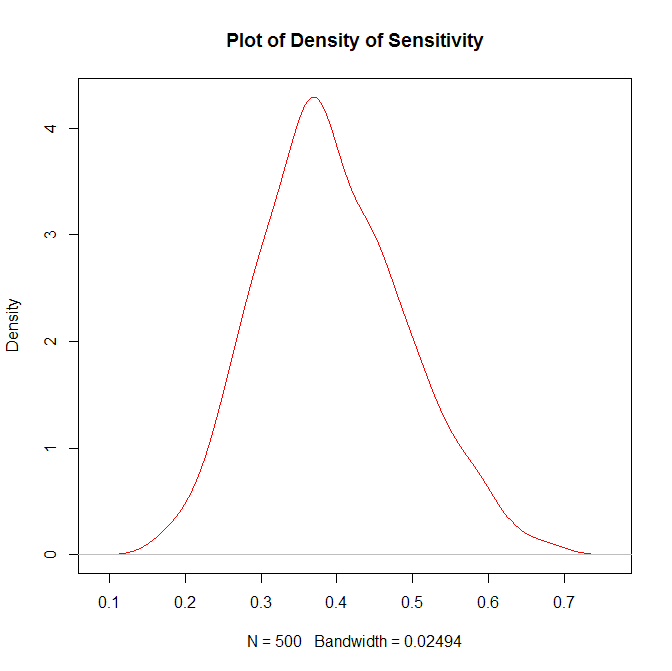* |
| --- | --- |
| 1. *Combination 6:* C_1_ΛC_3_=(C_1_ΛC_2_ΛC_3_)V(C_1_Λ¬C_2_ΛC_3_)   *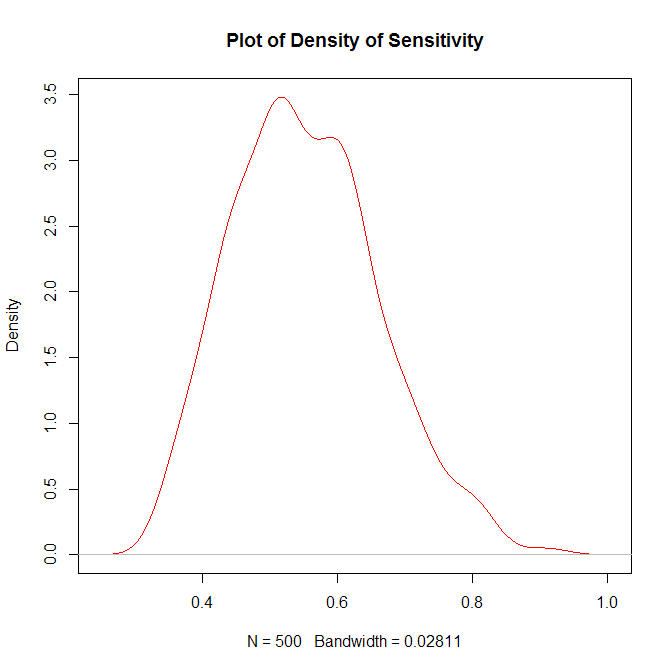* | 1. *Combination 18:* C_1_ΛC_2_=(C_1_ΛC_2_ΛC_3_)V(C_1_ΛC_2_Λ¬C_3_)   *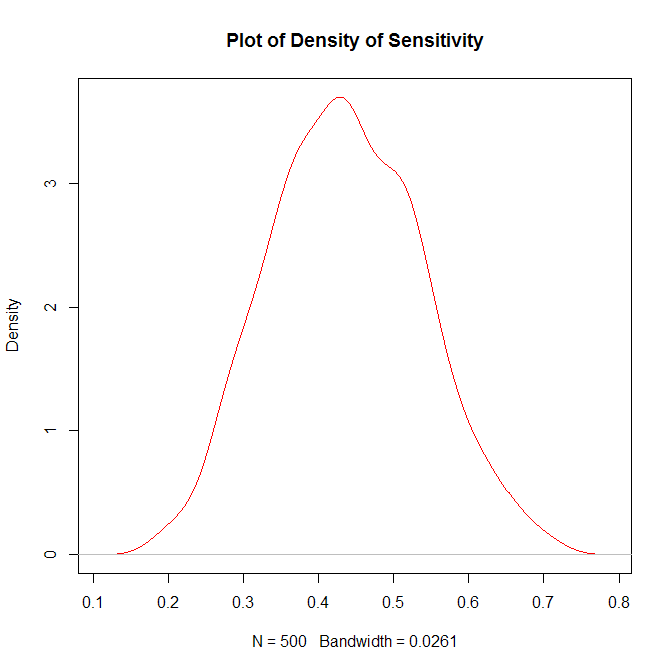* |

| 1. *Combination 24:* (C_1_ΛC_2_ΛC_3_)V(¬C_1_ΛC_2_ΛC_3_)V(C_1_Λ¬C_2_ΛC_3_)V (C_1_ΛC_2_Λ¬C_3_) *(At least two classifiers give a positive result)*   *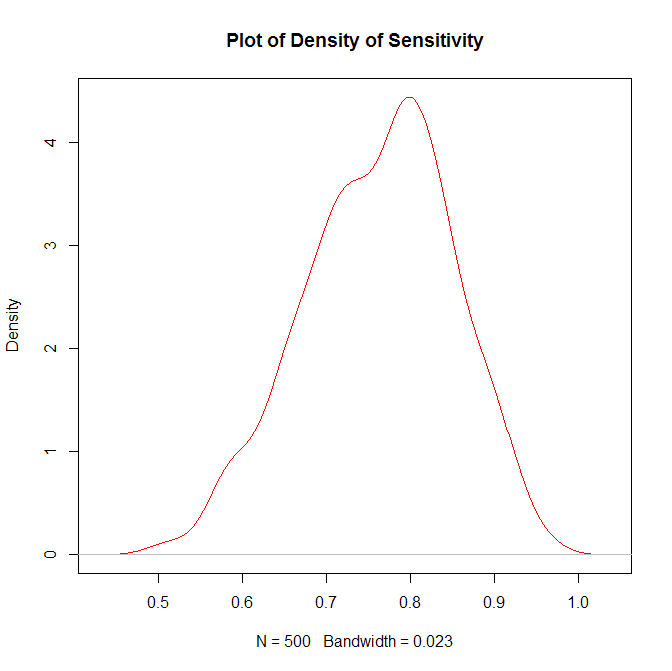* | 1. *Combination 64:* (C_1_ΛC_2_ΛC_3_)V(¬C_1_ΛC_2_ΛC_3_)V(C_1_Λ¬C_2_ΛC_3_)V (¬C_1_Λ¬C_2_ΛC_3_)V(C_1_ΛC_2_Λ¬C_3_)V (¬C_1_ΛC_2_Λ¬C_3_) C_2_VC_3_   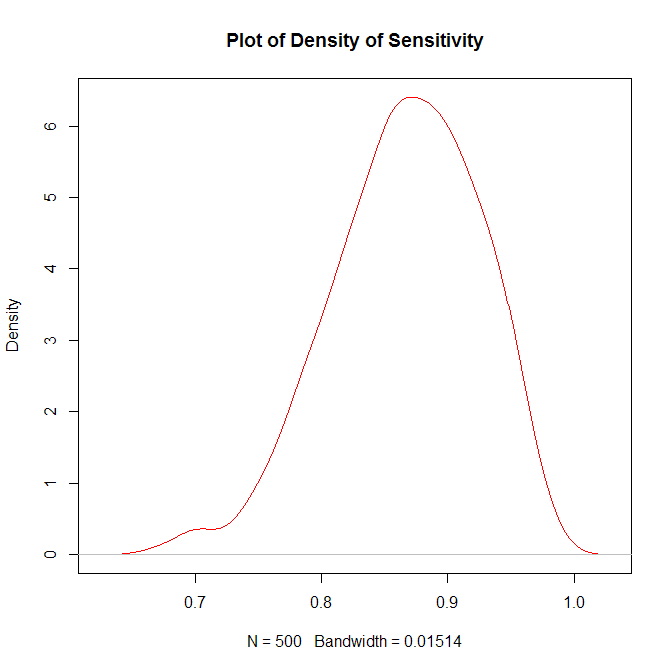 |
| --- | --- |
| 1. *Combination 96*^4^*:* (C_1_ΛC_2_ΛC_3_)V(¬C_1_ΛC_2_ΛC_3_)V(C_1_Λ¬C_2_ΛC_3_)V (¬C_1_Λ¬C_2_ΛC_3_)V(C_1_ΛC_2_Λ¬C_3_)V (C_1_Λ¬C_2_Λ¬C_3_) = C_1_VC_3_   *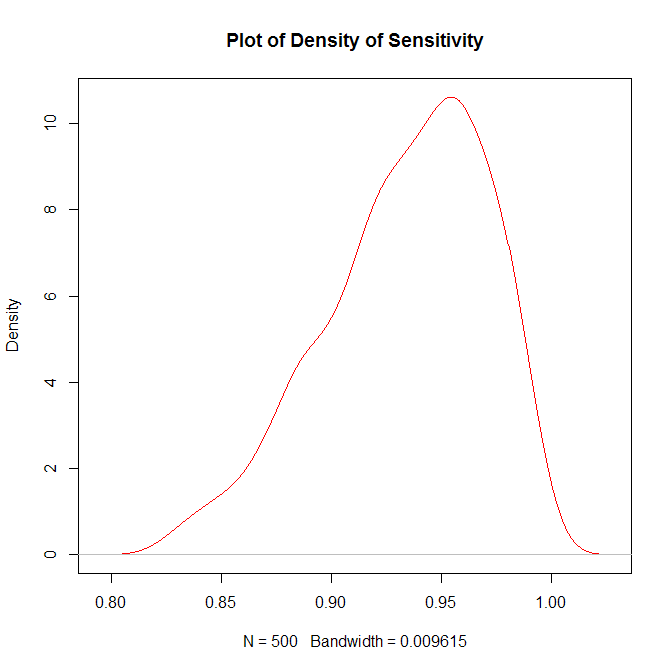* | 1. *Combination 120:* (C_1_ΛC_2_ΛC_3_)V(¬C_1_ΛC_2_ΛC_3_)V(C_1_Λ¬C_2_ΛC_3_)V (C_1_ΛC_2_Λ¬C_3_)V(¬C_1_ΛC_2_Λ¬C_3_)V (C_1_Λ¬C_2_Λ¬C_3_) = C_1_VC_2_   *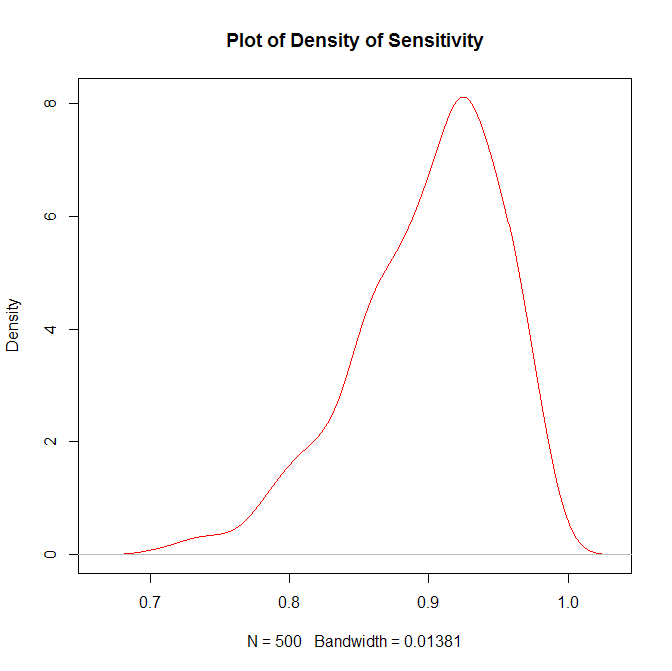* |

| 1. *Combination 128*^1,2,3^*:* C_1_VC_2_VC_3_= (C_1_ΛC_2_ΛC_3_)V(¬C_1_ΛC_2_ΛC_3_)V(C_1_Λ¬C_2_ΛC_3_)V(¬C_1_Λ¬C_2_ΛC_3_)V(C_1_ΛC_2_Λ¬C_3_)V(¬C_1_ΛC_2_Λ¬C_3_)V (C_1_Λ¬C_2_Λ¬C_3_)   **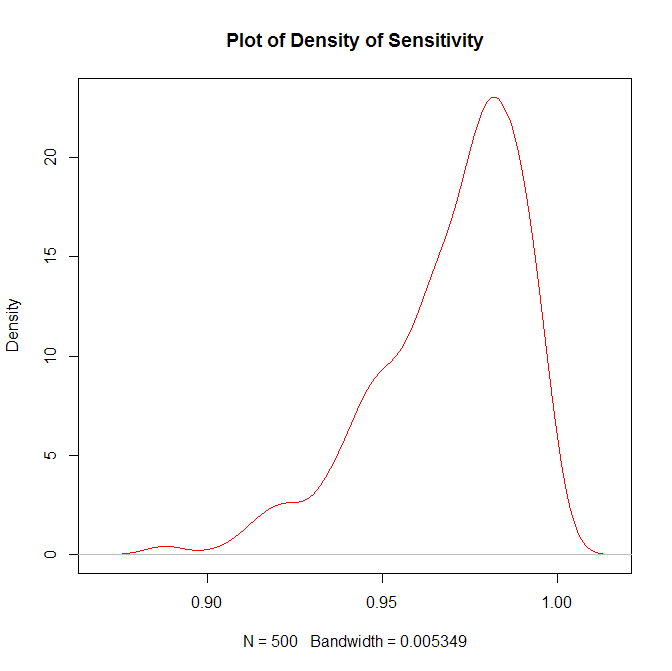** |
| --- |

^1^ Best combination according to ranking criterion 1 ^2^ Best combination according to ranking criterion 2

^3^ Best combination according to ranking criterion 3 ^4^ Best combination according to ranking criterion 4

**S7.1.2. Specificities of combinat**ions

| 1. *Combination 2: C_1_ΛC_2_ΛC_3_*   *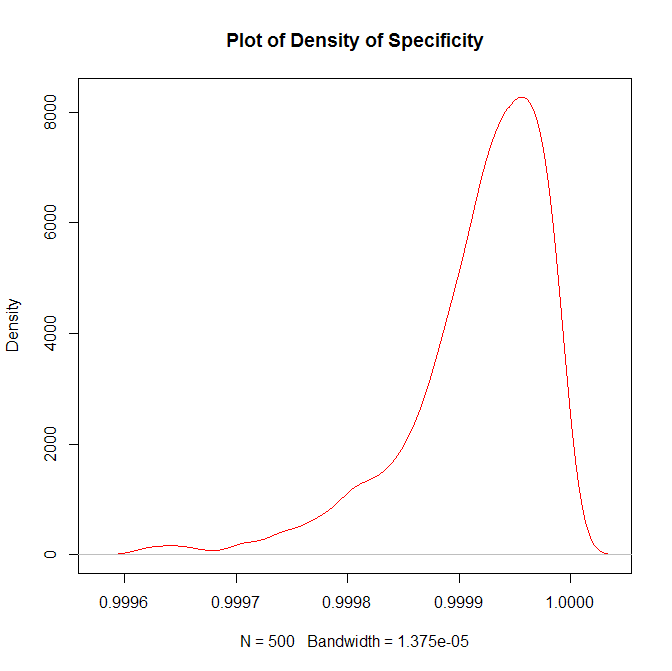* | 1. *Combination 4:* C_2_ΛC_3_=(C_1_ΛC_2_ΛC_3_)V(¬C_1_ΛC_2_ΛC_3_)   *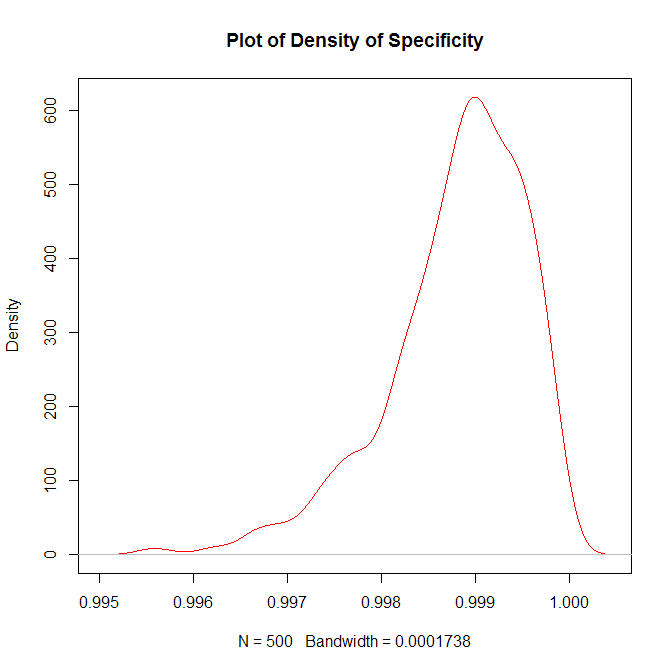* |
| --- | --- |
| 1. *Combination 6:* C_1_ΛC_3_=(C_1_ΛC_2_ΛC_3_)V(C_1_Λ¬C_2_ΛC_3_)   *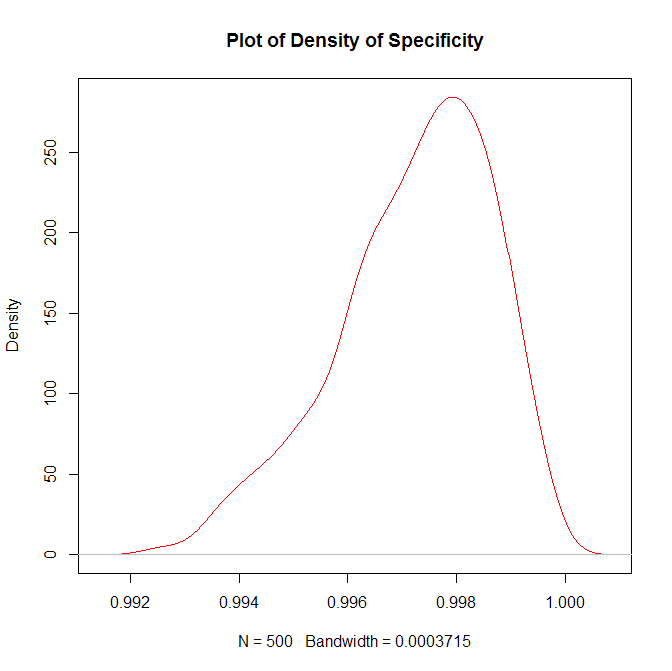* | 1. *Combination 18:* C_1_ΛC_2_=(C_1_ΛC_2_ΛC_3_)V(C_1_ΛC_2_Λ¬C_3_)   *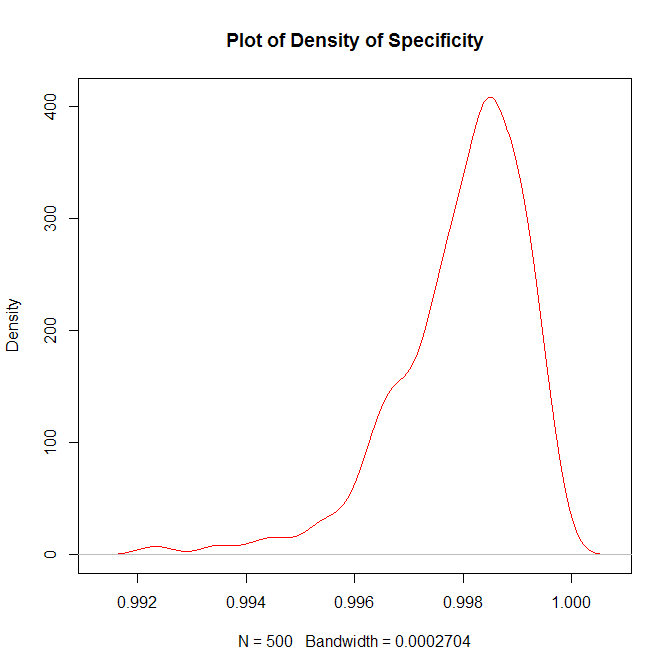* |

| 1. *Combination 24:* (C_1_ΛC_2_ΛC_3_)V(¬C_1_ΛC_2_ΛC_3_)V(C_1_Λ¬C_2_ΛC_3_)V (C_1_ΛC_2_Λ¬C_3_) *(At least two classifiers give a positive result)*   *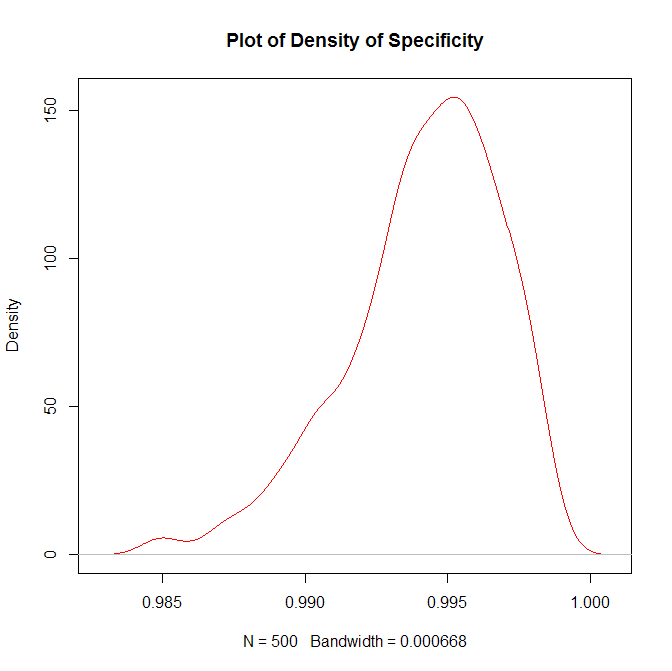* | 1. *Combination 64:* (C_1_ΛC_2_ΛC_3_)V(¬C_1_ΛC_2_ΛC_3_)V(C_1_Λ¬C_2_ΛC_3_)V (¬C_1_Λ¬C_2_ΛC_3_)V(C_1_ΛC_2_Λ¬C_3_)V (¬C_1_ΛC_2_Λ¬C_3_) =C_2_VC_3_   *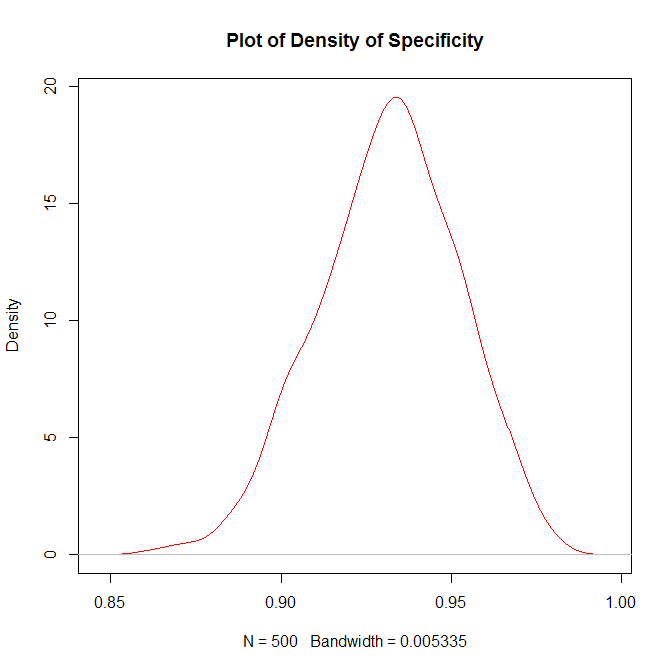* |
| --- | --- |
| 1. *Combination 96*^4^*:* (C_1_ΛC_2_ΛC_3_)V(¬C_1_ΛC_2_ΛC_3_)V(C_1_Λ¬C_2_ΛC_3_)V (¬C_1_Λ¬C_2_ΛC_3_)V(C_1_ΛC_2_Λ¬C_3_)V (C_1_Λ¬C_2_Λ¬C_3_) = C_1_VC_3_   *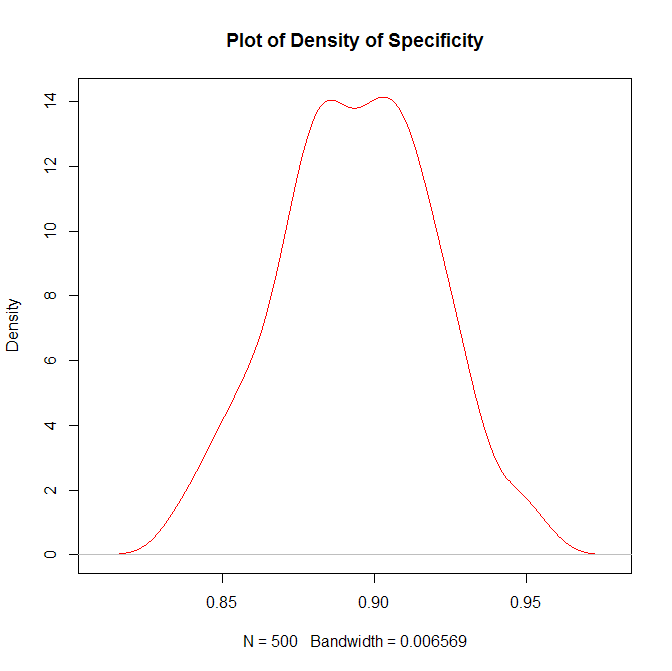* | 1. *Combination 120:* (C_1_ΛC_2_ΛC_3_)V(¬C_1_ΛC_2_ΛC_3_)V(C_1_Λ¬C_2_ΛC_3_)V (C_1_ΛC_2_Λ¬C_3_)V(¬C_1_ΛC_2_Λ¬C_3_)V (C_1_Λ¬C_2_Λ¬C_3_) = C_1_VC_2_   *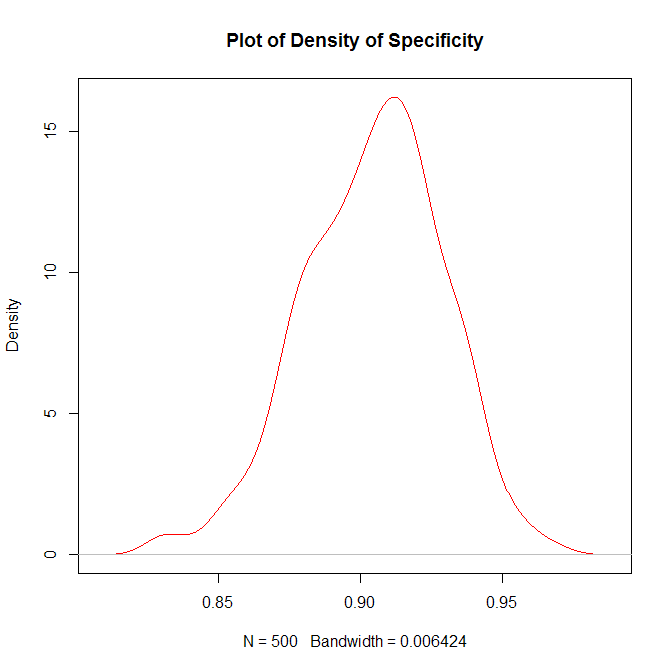* |

| 1. *Combination 128*^1,2,3^*:* C_1_VC_2_VC_3_= (C_1_ΛC_2_ΛC_3_)V(¬C_1_ΛC_2_ΛC_3_)V(C_1_Λ¬C_2_ΛC_3_)V(¬C_1_Λ¬C_2_ΛC_3_)V(C_1_ΛC_2_Λ¬C_3_)V(¬C_1_ΛC_2_Λ¬C_3_)V (C_1_Λ¬C_2_Λ¬C_3_)   **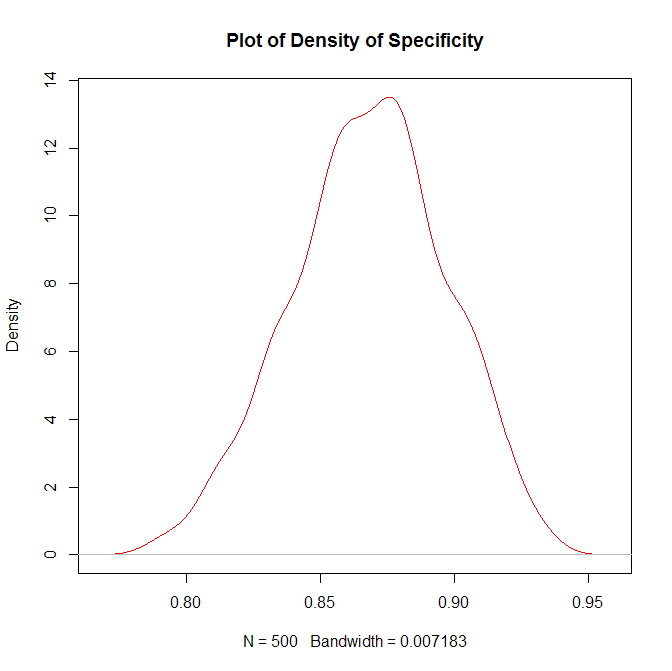** |
| --- |

^1^ Best combination according to ranking criterion 1 ^2^ Best combination according to ranking criterion 2

^3^ Best combination according to ranking criterion 3 ^4^ Best combination according to ranking criterion 4

S7.1.3. Summary statistics of ranking methods

|  | Method 1 |  | Method 2 |  | Method 3 |  | Method 4 |  |
| --- | --- | --- | --- | --- | --- | --- | --- | --- |
| Combination | Mean | SD | Mean | SD | Mean | SD | Mean | SD |
| *C_1_ΛC_2_ΛC_3_* | 0.311 | 0.094 | 1.105 | 0.064 | 1.311 | 0.094 | 0.311 | 0.094 |
| C_2_ΛC_3_ | 0.394 | 0.096 | 1.162 | 0.079 | 1.393 | 0.096 | 0.394 | 0.096 |
| C_1_ΛC_3_ | 0.551 | 0.108 | 1.312 | 0.124 | 1.550 | 0.108 | 0.553 | 0.108 |
| C_1_ΛC_2_ | 0.435 | 0.100 | 1.196 | 0.089 | 1.434 | 0.100 | 0.436 | 0.101 |
| At least two | 0.757 | 0.087 | 1.576 | 0.130 | 1.756 | 0.087 | 0.762 | 0.089 |
| C_2_VC_3_ | 0.807 | 0.051 | 1.623 | 0.093 | 1.798 | 0.055 | 0.861 | 0.052 |
| C_1_VC_3_^4^ | 0.835 | 0.033 | 1.674 | 0.067 | 1.828 | 0.037 | 0.887 | 0.023 |
| C_1_VC_2_ | 0.814 | 0.049 | 1.631 | 0.096 | 1.804 | 0.055 | 0.877 | 0.039 |
| C_1_VC_2_VC_3_^1,2,3^ | 0.841 | 0.025 | 1.694 | 0.045 | 1.837 | 0.026 | 0.868 | 0.028 |

^1^ Best combination according to ranking criterion 1 ^2^ Best combination according to ranking criterion 2

^3^ Best combination according to ranking criterion 3 ^4^ Best combination according to ranking criterion 4

**S7.2. Swine flu**

**S7.2.1. Sensitivities** of combinations

| 1. *Combination 1:* All results are negative   This combination is trivial. It classes everything as negative, so it will always have a sensitivity of 0. | 1. *Combination 2:* C_1_ΛC_2_   *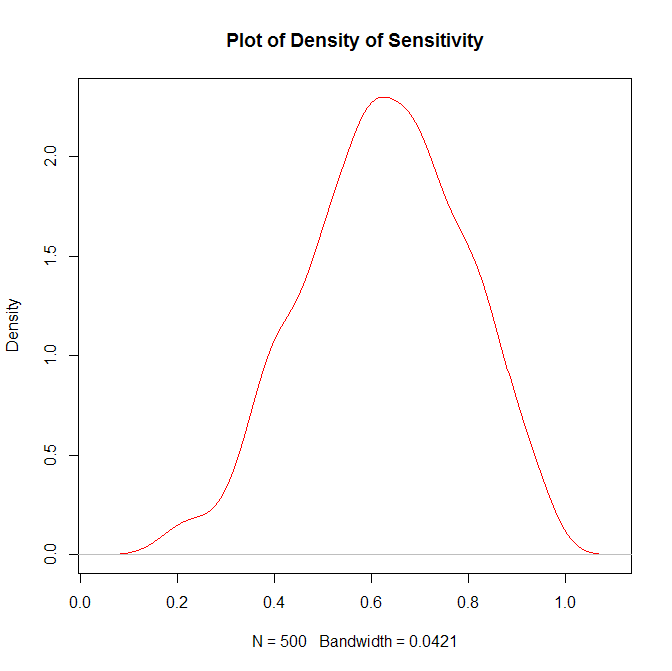* |
| --- | --- |
| 1. *Combination 3:* ¬C_1_ΛC_2_   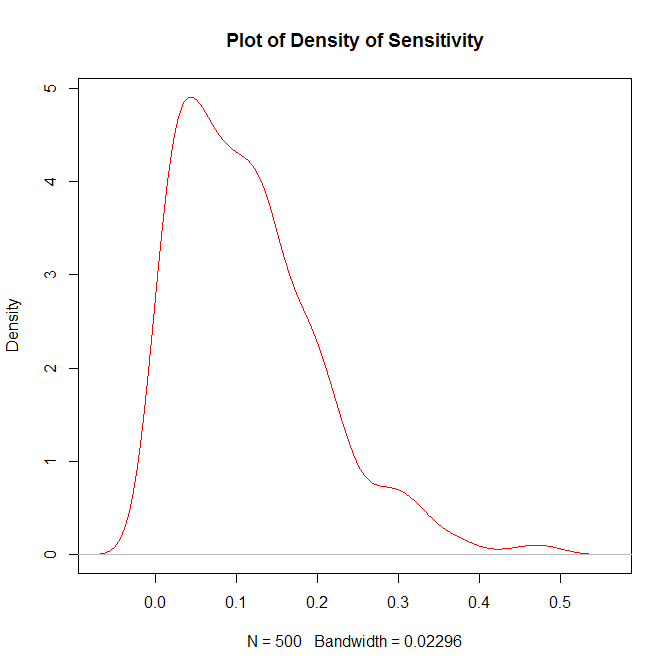 | 1. *Combination 4:* (C_1_ΛC_2_)V(¬C_1_ΛC_2_)=C_2_   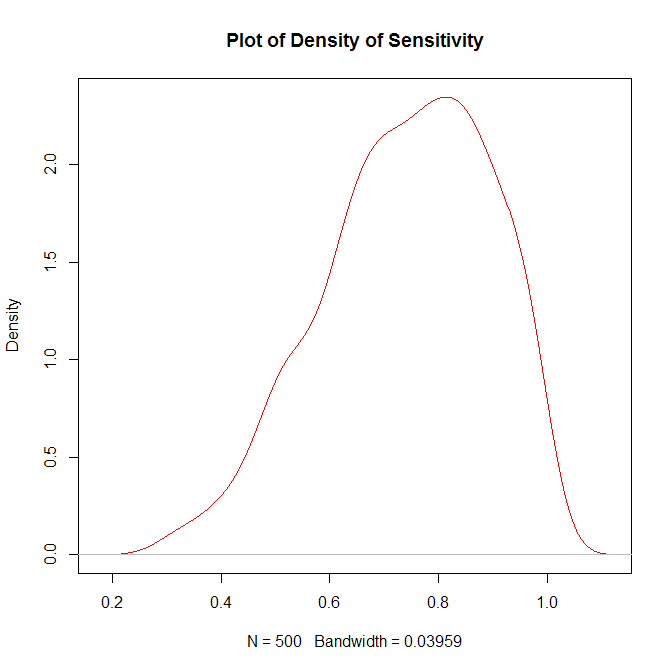 |
| 1. *Combination 5:* C_1_Λ¬C_2_   *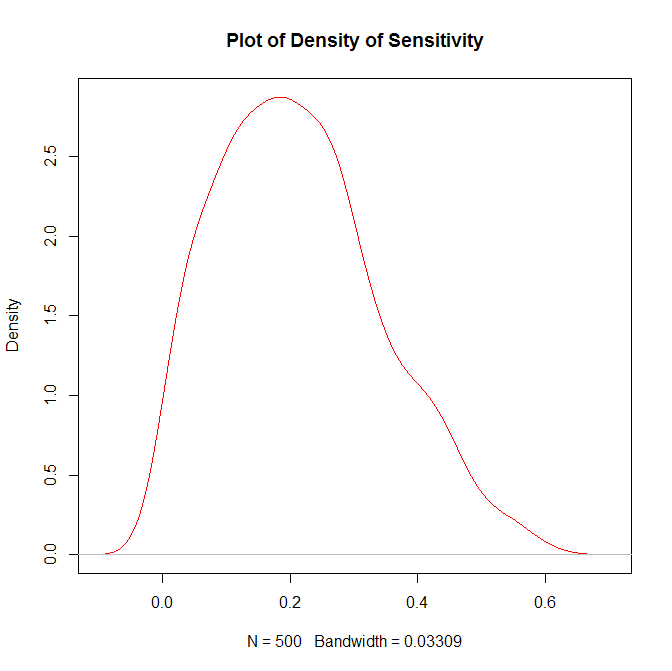* | 1. *Combination 6*^4^*:* (C_1_ΛC_2_)V(C_1_Λ¬C_2_)=C_1_   *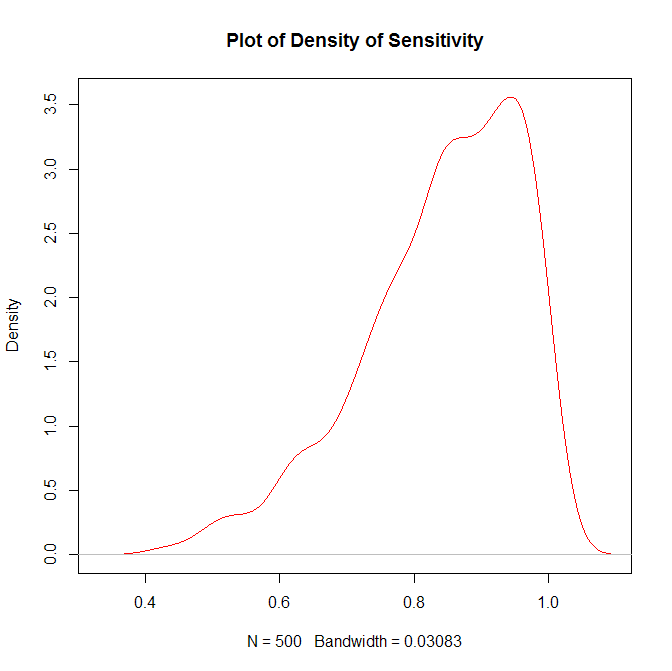* |

| 1. *Combination 7:* (¬C_1_ΛC_2_)V(C_1_Λ¬C_2_)   *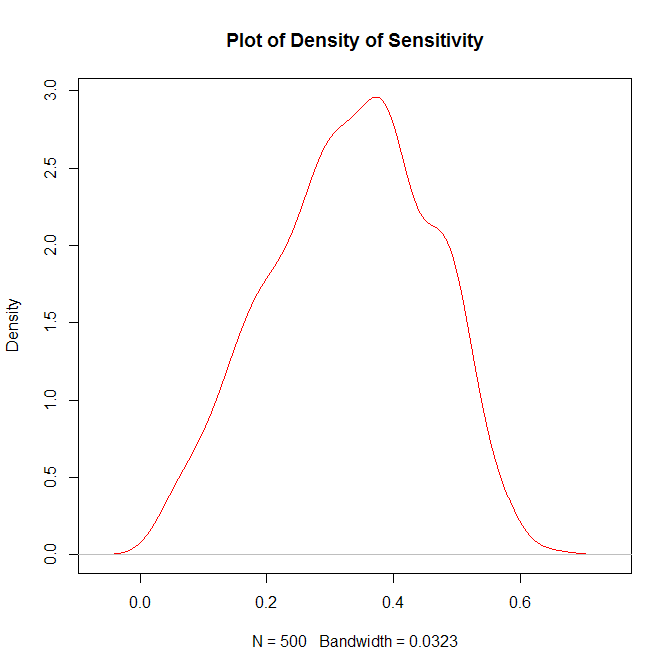* | 1. *Combination 8*^1,2,3^*:* (C_1_ΛC_2_)V(¬C_1_ΛC_2_)V(C_1_Λ¬C_2_)=C_1_VC_2_   *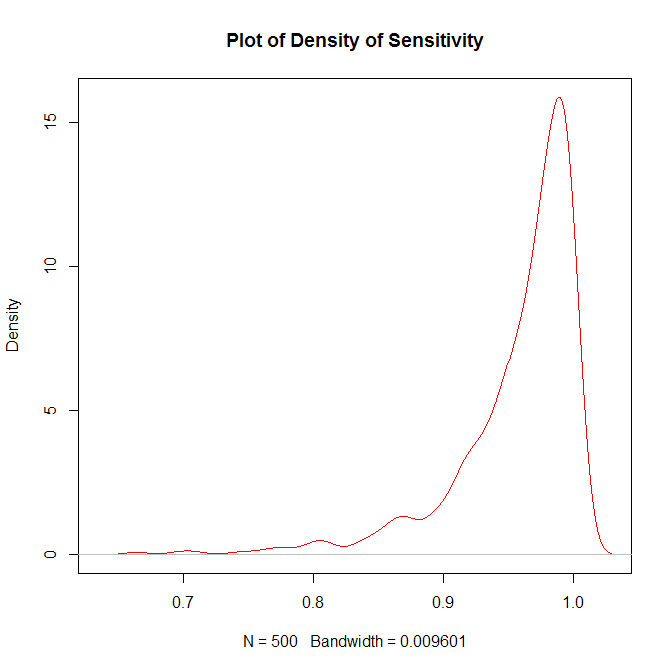* |
| --- | --- |
| 1. *Combination 9:* ¬C_1_Λ¬C_2_=¬(C_1_ΛC_2_)   *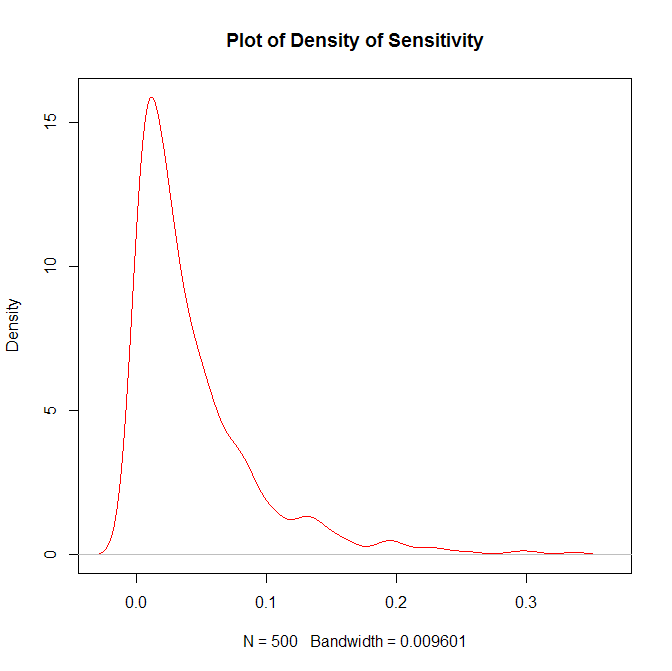* | 1. *Combination 10:* (C_1_ΛC_2_)V(¬C_1_Λ¬C_2_)   *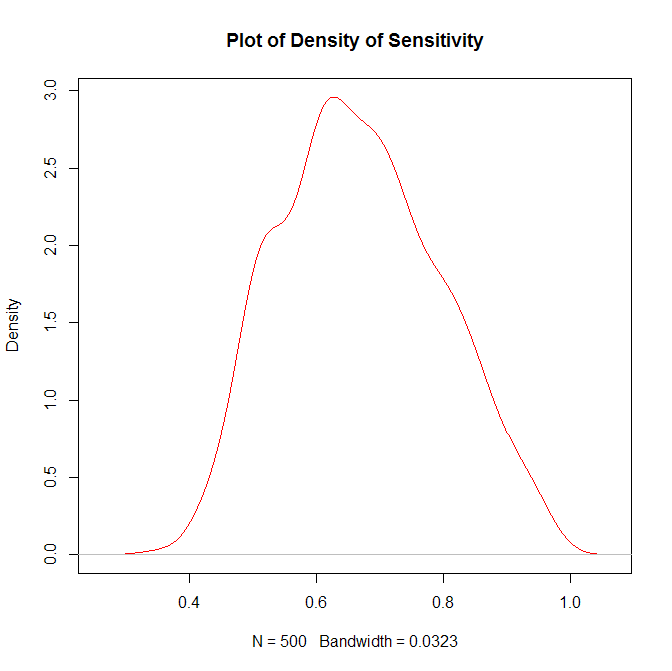* |
| 1. *Combination 11:* (¬C_1_ΛC_2_)V(¬C_1_Λ¬C_2_)   *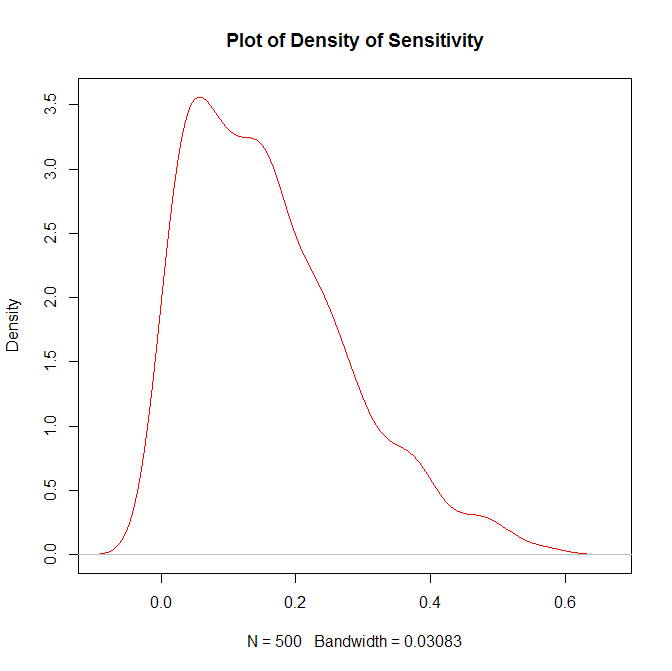* | 1. *Combination 12:* (C_1_ΛC_2_)V(¬C_1_ΛC_2_)V(¬C_1_Λ¬C_2_)=¬(C_1_Λ¬C_2_)   *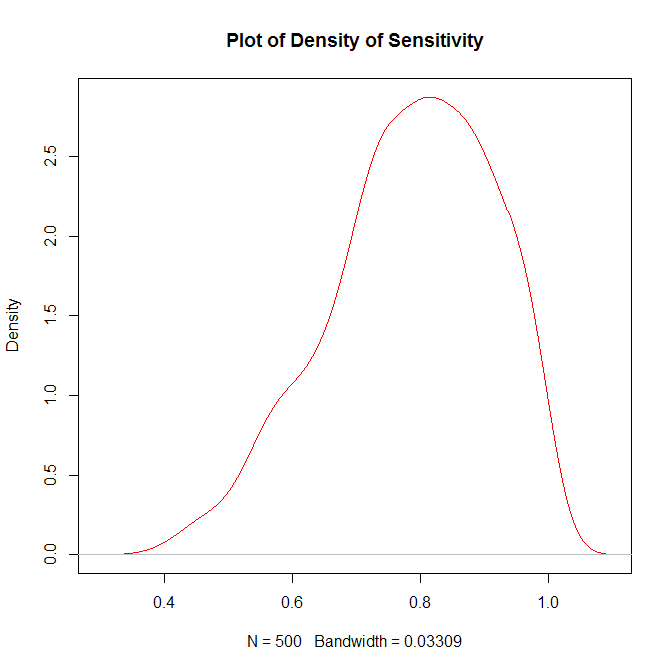* |

| 1. *Combination 13:* (C_1_Λ¬C_2_)V(¬C_1_Λ¬C_2_)= ¬C_2_   *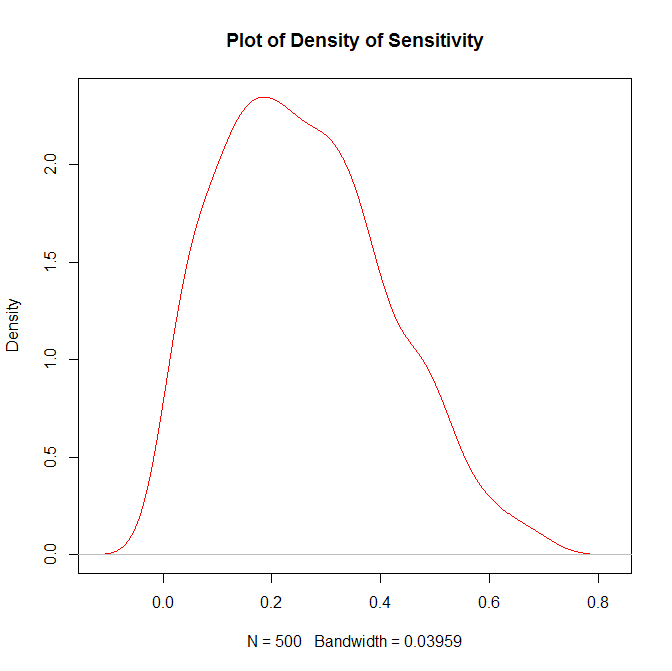* | 1. *Combination 14:* (C_1_ΛC_2_)V(C_1_Λ¬C_2_)V(¬C_1_Λ¬C_2_)   *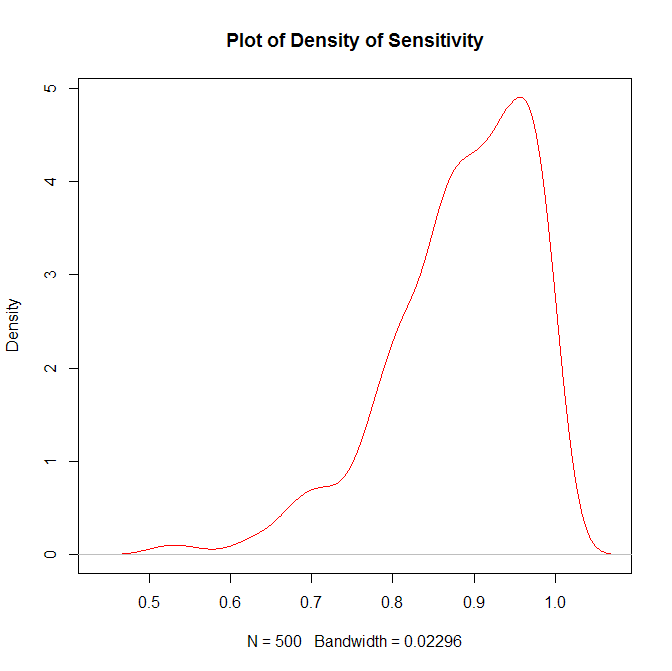* |
| --- | --- |
| 1. *Combination 15:* (¬C_1_ΛC_2_)V(C_1_Λ¬C_2_)V(¬C_1_Λ¬C_2_)   *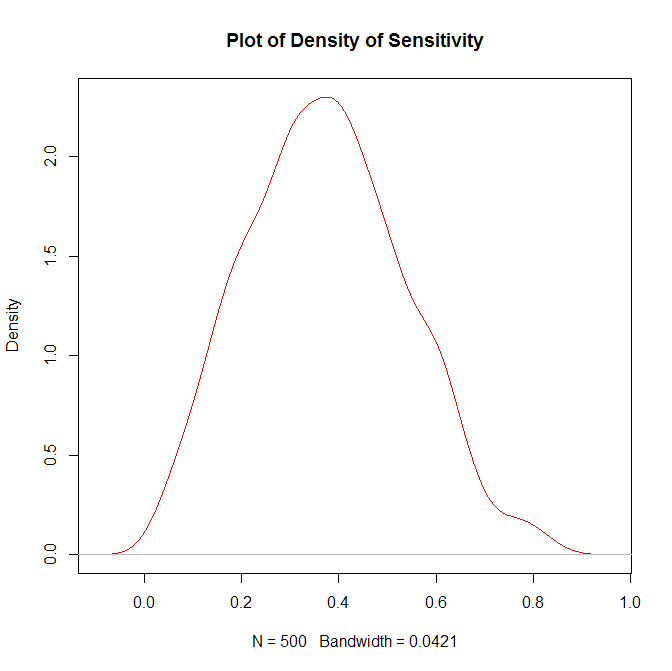* | 1. *Combination 16:* (C_1_ΛC_2_)V(¬C_1_ΛC_2_)V(C_1_Λ¬C_2_)V(¬C_1_Λ¬C_2_)   (All results are positive)  This combination is trivial. It classes everything as positive, so it will always have a sensitivity of 1. |

^1^ Best combination according to ranking criterion 1 ^2^ Best combination according to ranking criterion 2

^3^ Best combination according to ranking criterion 3 ^4^ Best combination according to ranking criterion 4

S7.2.2. Specificities of combinations

| 1. *Combination 1:* All results are negative   This combination is trivial. It classes everything as negative, so it will always have a specificity of 1. | 1. *Combination 2:* C_1_ΛC_2_   *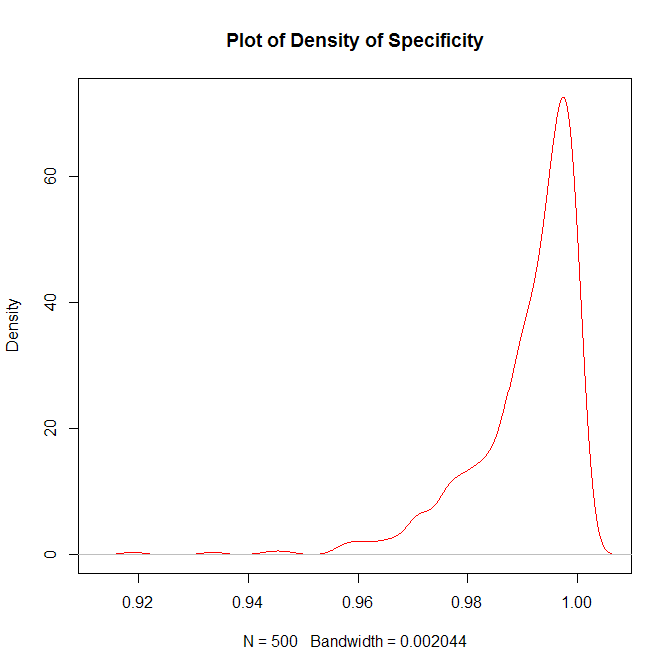* |
| --- | --- |
| 1. *Combination 3:* ¬C_1_ΛC_2_   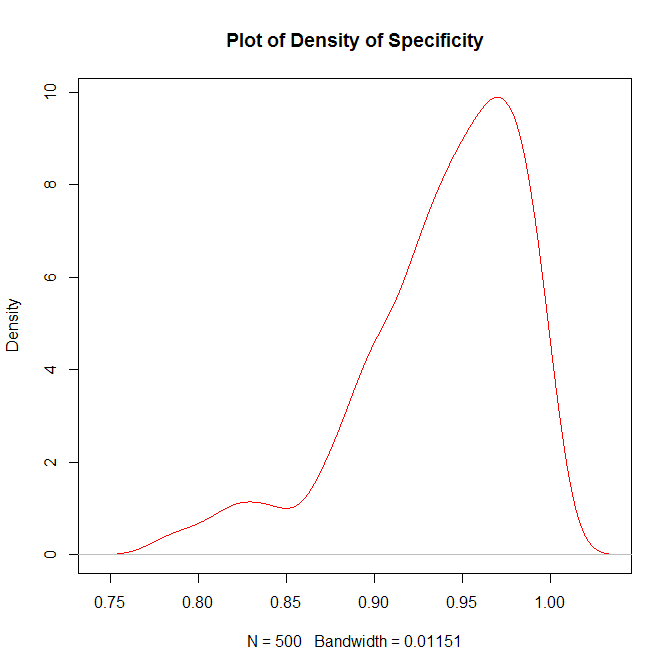 | 1. *Combination 4:* (C_1_ΛC_2_)V(¬C_1_ΛC_2_)=C_2_   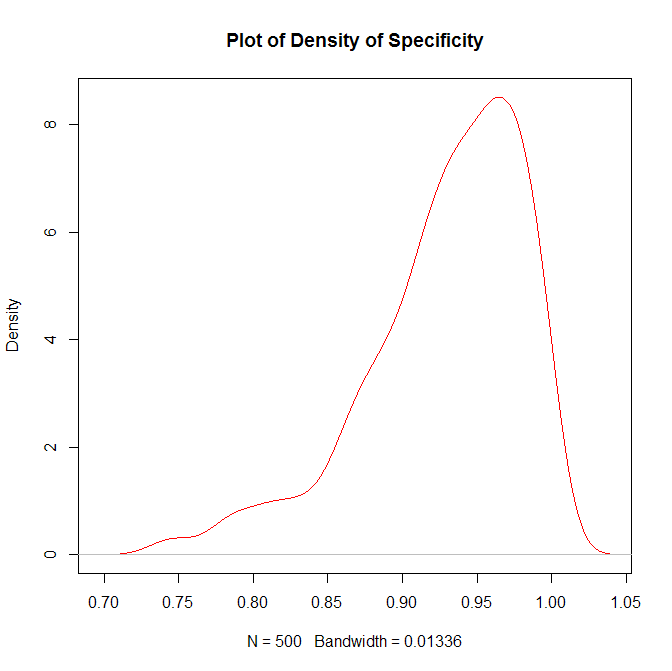 |
| 1. *Combination 5:* C_1_Λ¬C_2_   *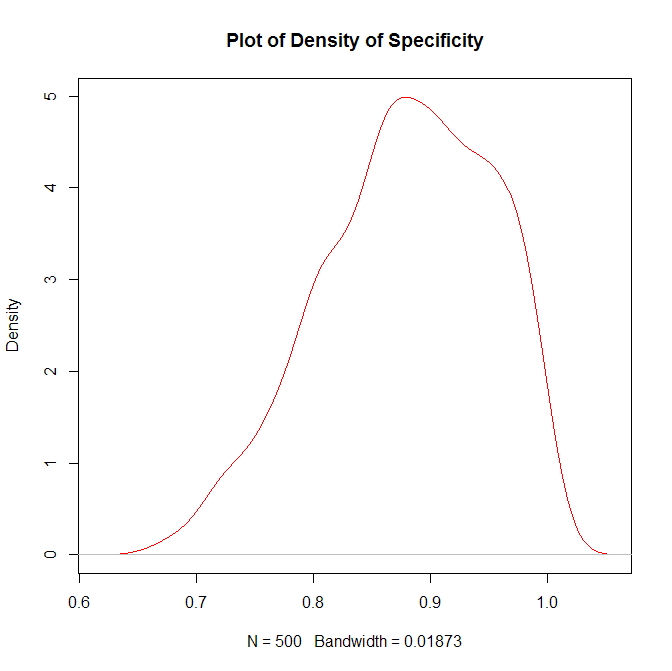* | 1. *Combination 6*^4^*:* (C_1_ΛC_2_)V(C_1_Λ¬C_2_)=C_1_   *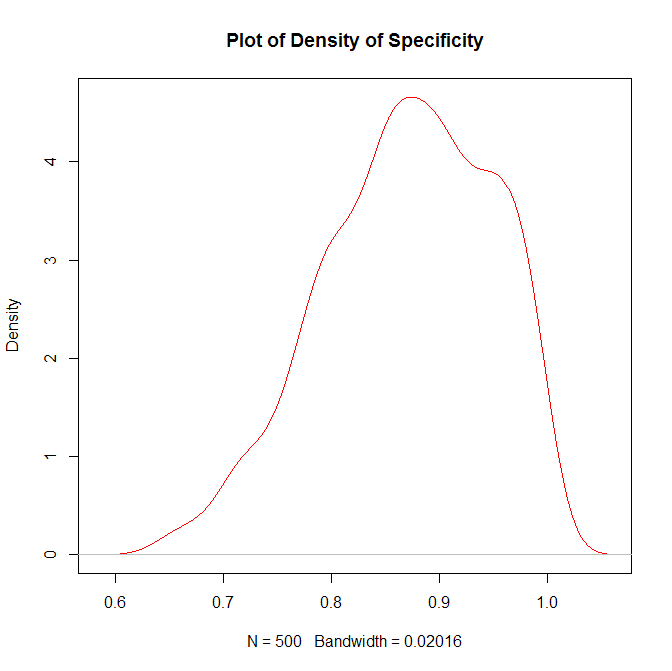* |

| 1. *Combination 7:* (¬C_1_ΛC_2_)V(C_1_Λ¬C_2_)   *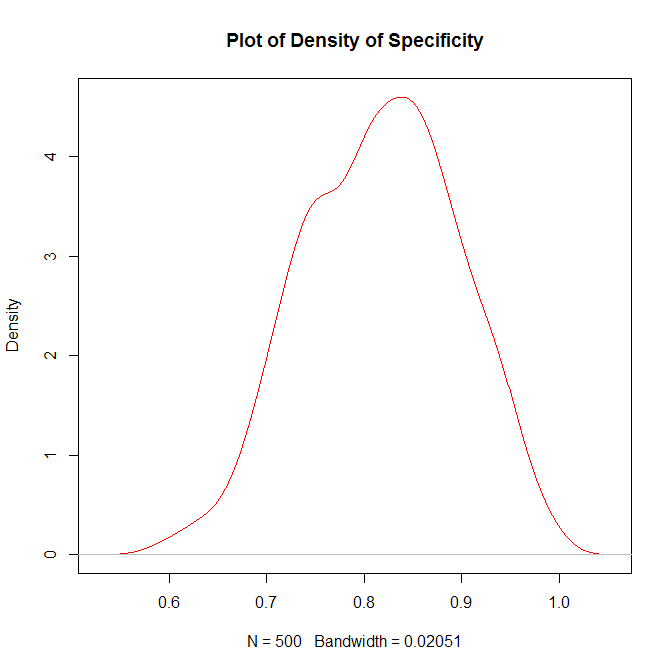* | 1. *Combination 8*^1,2,3^*:* (C_1_ΛC_2_)V(¬C_1_ΛC_2_)V(C_1_Λ¬C_2_)=C_1_VC_2_   *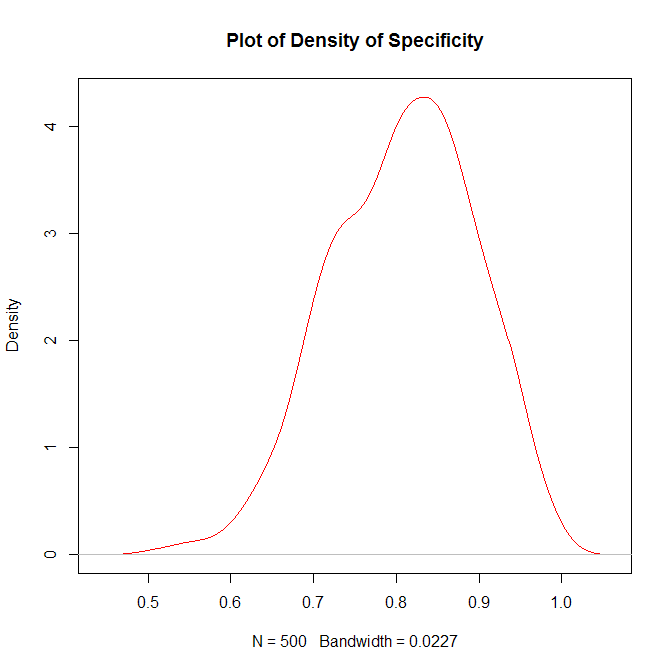* |
| --- | --- |
| 1. *Combination 9:* ¬C_1_Λ¬C_2_=¬(C_1_ΛC_2_)   *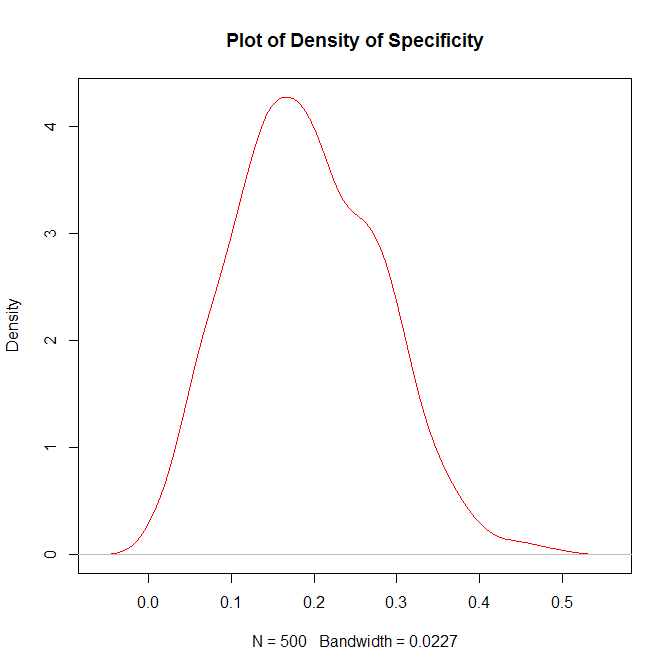* | 1. *Combination 10:* (C_1_ΛC_2_)V(¬C_1_Λ¬C_2_)   *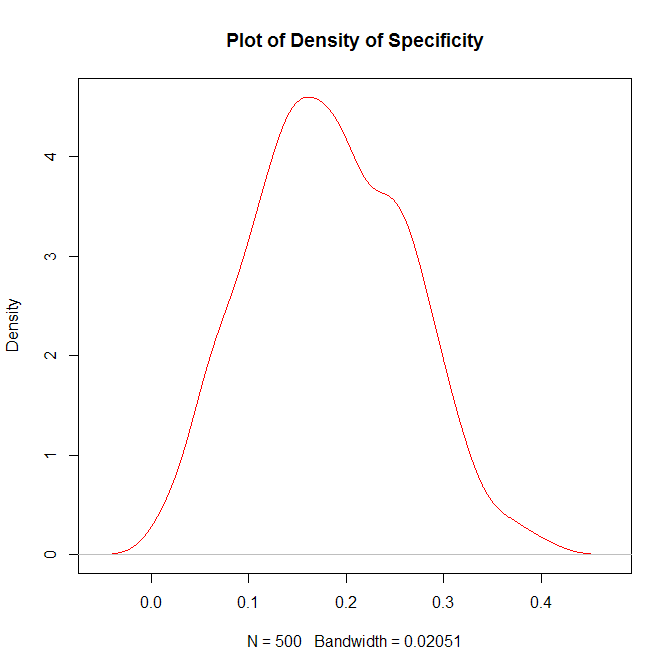* |
| 1. *Combination 11:* (¬C_1_ΛC_2_)V(¬C_1_Λ¬C_2_)   *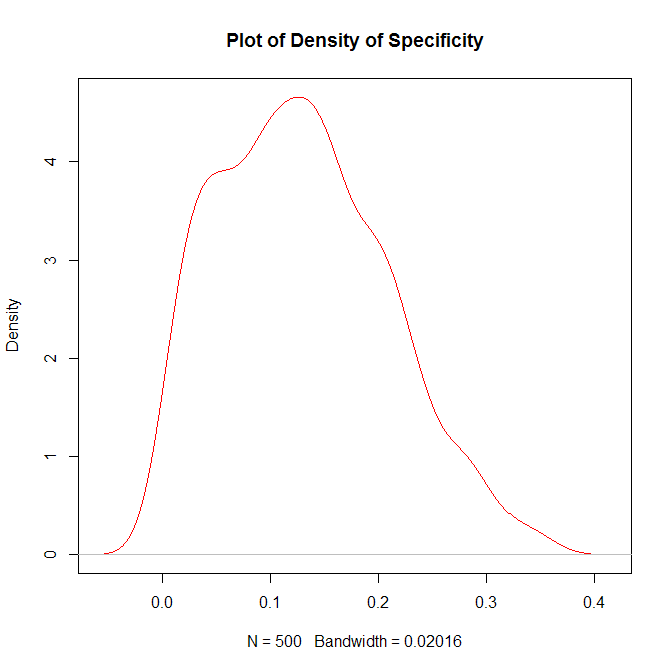* | 1. *Combination 12:* (C_1_ΛC_2_)V(¬C_1_ΛC_2_)V(¬C_1_Λ¬C_2_)=¬(C_1_Λ¬C_2_)   *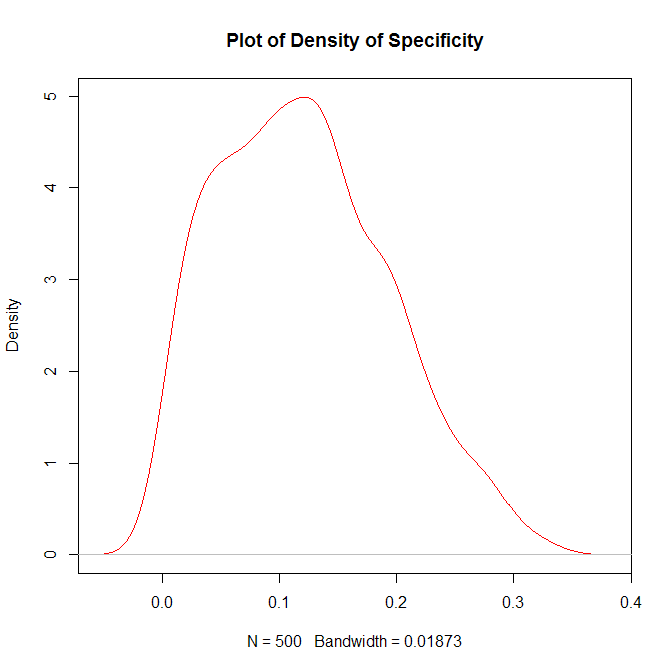* |
| 1. *Combination 13:* (C_1_Λ¬C_2_)V(¬C_1_Λ¬C_2_)= ¬C_2_   *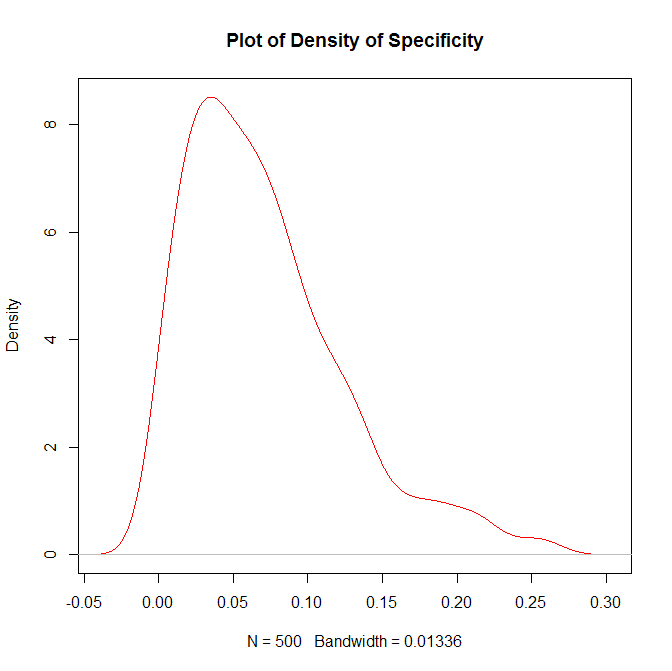* | 1. *Combination 14:* (C_1_ΛC_2_)V(C_1_Λ¬C_2_)V(¬C_1_Λ¬C_2_)   *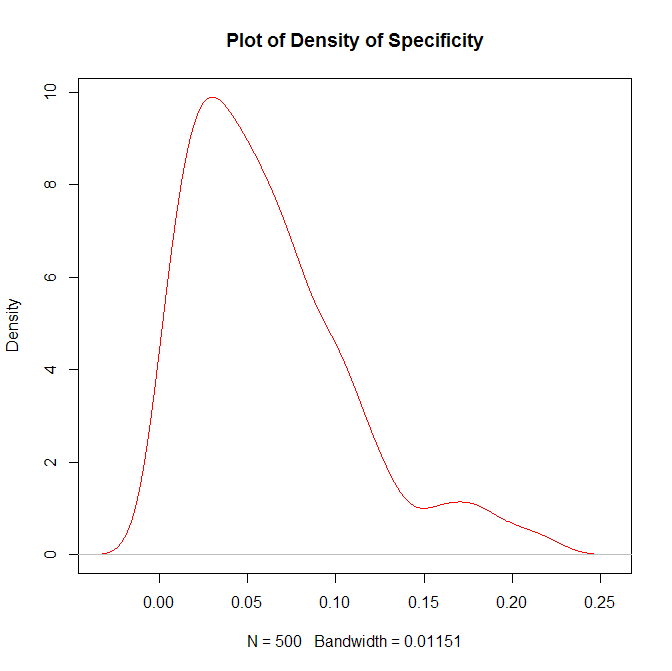* |
| 1. *Combination 15:* (¬C_1_ΛC_2_)V(C_1_Λ¬C_2_)V(¬C_1_Λ¬C_2_)   *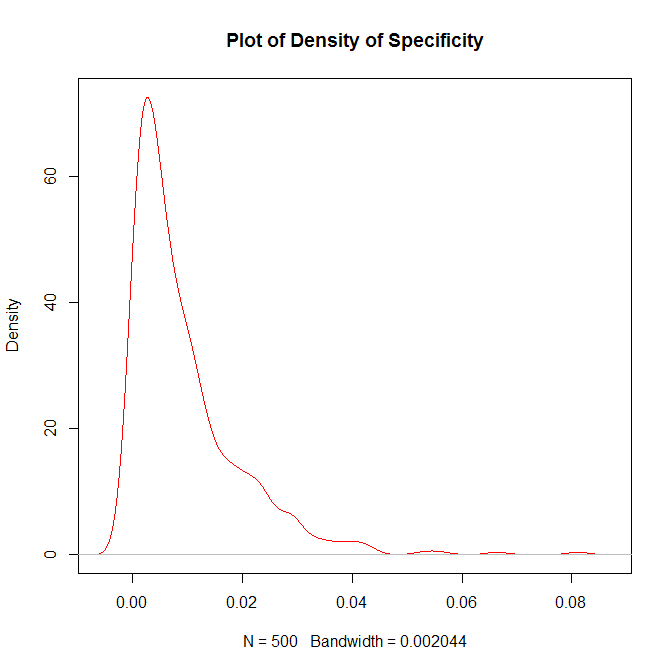* | 1. *Combination 16:* (C_1_ΛC_2_)V(¬C_1_ΛC_2_)V(C_1_Λ¬C_2_)V(¬C_1_Λ¬C_2_)   (All results are positive)  This combination is trivial. It classes everything as negative, so it will always have a specificity of 0. |

^1^ Best combination according to ranking criterion 1 ^2^ Best combination according to ranking criterion 2

^3^ Best combination according to ranking criterion 3 ^4^ Best combination according to ranking criterion 4

S7.2.3. Summary statistics of ranking methods

|  | Method 1 |  | Method 2 |  | Method 3 |  | Method 4 |  |
| --- | --- | --- | --- | --- | --- | --- | --- | --- |
| Combination | Mean | SD | Mean | SD | Mean | SD | Mean | SD |
| All negative | 0.000 | 0.000 | 1.000 | 0.000 | 1.000 | 0.000 | 0.000 | 0.000 |
| C_1_ΛC_2_ | 0.620 | 0.160 | 1.400 | 0.198 | 1.617 | 0.161 | 0.626 | 0.162 |
| ¬C_1_ΛC_2_ | 0.110 | 0.085 | 0.903 | 0.097 | 1.054 | 0.110 | 0.116 | 0.088 |
| C_2_ | 0.690 | 0.152 | 1.439 | 0.250 | 1.671 | 0.168 | 0.737 | 0.147 |
| C_1_Λ¬C_2_ | 0.192 | 0.121 | 0.841 | 0.161 | 1.094 | 0.170 | 0.214 | 0.127 |
| C_1_^4^ | 0.731 | 0.123 | 1.483 | 0.231 | 1.710 | 0.142 | 0.798 | 0.100 |
| (¬C_1_ΛC_2_)V(C_1_Λ¬C_2_) | 0.273 | 0.112 | 0.799 | 0.170 | 1.148 | 0.164 | 0.330 | 0.124 |
| C_1_VC_2_^1,2,3^ | 0.772 | 0.086 | 1.578 | 0.151 | 1.764 | 0.093 | 0.803 | 0.083 |
| ¬(C_1_ΛC_2_) | 0.008 | 0.011 | 0.049 | 0.039 | 0.236 | 0.093 | 0.039 | 0.042 |
| (C_1_ΛC_2_)V(¬C_1_Λ¬C_2_) | 0.125 | 0.063 | 0.503 | 0.180 | 0.852 | 0.164 | 0.183 | 0.079 |
| (¬C_1_ΛC_2_)V(¬C_1_Λ¬C_2_) | 0.021 | 0.022 | 0.063 | 0.059 | 0.290 | 0.142 | 0.088 | 0.064 |
| ¬(C_1_Λ¬C_2_) | 0.098 | 0.065 | 0.654 | 0.201 | 0.906 | 0.170 | 0.121 | 0.072 |
| ¬C_2_ | 0.020 | 0.022 | 0.098 | 0.095 | 0.329 | 0.168 | 0.067 | 0.051 |
| (C_1_ΛC_2_)V(C_1_Λ¬C_2_)  V(¬C_1_Λ¬C_2_) | 0.056 | 0.044 | 0.795 | 0.150 | 0.946 | 0.110 | 0.062 | 0.047 |
| (¬C_1_ΛC_2_)V(C_1_Λ¬C_2_)  V(¬C_1_Λ¬C_2_) | 0.003 | 0.004 | 0.166 | 0.131 | 0.383 | 0.161 | 0.009 | 0.010 |
| All positive | 0.000 | 0.000 | 1.000 | 0.000 | 1.000 | 0.000 | 0.000 | 0.000 |

^1^ Best combination according to ranking criterion 1 ^2^ Best combination according to ranking criterion 2

^3^ Best combination according to ranking criterion 3 ^4^ Best combination according to ranking criterion 4

**S7.3. SNP**

S7.3.1. Summary statistics of ranking methods - ~1000 positives

|  | Method 1 |  | Method 2 |  | Method 3 |  | Method 4 |  |
| --- | --- | --- | --- | --- | --- | --- | --- | --- |
| Combination | Mean | SD | Mean | SD | Mean | SD | Mean | SD |
| *C_1_ΛC_2_ΛC_3_* | 0.083 | 0.017 | 1.007 | 0.003 | 1.083 | 0.017 | 0.083 | 0.017 |
| C_2_ΛC_3_ | 0.098 | 0.018 | 1.010 | 0.004 | 1.098 | 0.018 | 0.098 | 0.018 |
| C_1_ΛC_3_ | 0.144 | 0.020 | 1.021 | 0.006 | 1.144 | 0.020 | 0.144 | 0.020 |
| C_1_ΛC_2_ | 0.483 | 0.053 | 1.236 | 0.052 | 1.483 | 0.053 | 0.483 | 0.053 |
| At least two | 0.559 | 0.053 | 1.315 | 0.060 | 1.559 | 0.053 | 0.559 | 0.053 |
| C_2_VC_3_ | 0.643 | 0.047 | 1.412 | 0.061 | 1.642 | 0.047 | 0.645 | 0.047 |
| C_1_VC_3_ | 0.866 | 0.034 | 1.750 | 0.060 | 1.866 | 0.035 | 0.869 | 0.035 |
| C_1_VC_2_ | 0.930 | 0.021 | 1.866 | 0.039 | 1.930 | 0.021 | 0.932 | 0.021 |
| C_1_VC_2_VC_3_^1,2,3,4^ | 0.940 | 0.018 | 1.883 | 0.034 | 1.940 | 0.018 | 0.943 | 0.018 |

^1^ Best combination according to ranking criterion 1 ^2^ Best combination according to ranking criterion 2

^3^ Best combination according to ranking criterion 3 ^4^ Best combination according to ranking criterion 4

**S7.3.2. Summary statistics of ranking methods** - ~5000 positives

|  | Method 1 |  | Method 2 |  | Method 3 |  | Method 4 |  |
| --- | --- | --- | --- | --- | --- | --- | --- | --- |
| Combination | Mean | SD | Mean | SD | Mean | SD | Mean | SD |
| *C_1_ΛC_2_ΛC_3_* | 0.065 | 0.007 | 1.004 | 0.001 | 1.065 | 0.007 | 0.065 | 0.007 |
| C_2_ΛC_3_ | 0.079 | 0.007 | 1.006 | 0.001 | 1.079 | 0.007 | 0.079 | 0.007 |
| C_1_ΛC_3_ | 0.123 | 0.008 | 1.015 | 0.002 | 1.123 | 0.008 | 0.123 | 0.008 |
| C_1_ΛC_2_ | 0.439 | 0.025 | 1.193 | 0.022 | 1.438 | 0.025 | 0.439 | 0.025 |
| At least two | 0.510 | 0.026 | 1.260 | 0.026 | 1.510 | 0.026 | 0.510 | 0.026 |
| C_2_VC_3_ | 0.594 | 0.023 | 1.337 | 0.029 | 1.589 | 0.024 | 0.601 | 0.024 |
| C_1_VC_3_ | 0.843 | 0.018 | 1.705 | 0.030 | 1.841 | 0.018 | 0.850 | 0.018 |
| C_1_VC_2_ | 0.912 | 0.011 | 1.830 | 0.020 | 1.912 | 0.011 | 0.918 | 0.011 |
| C_1_VC_2_VC_3_^1,2,3,4^ | 0.917 | 0.010 | 1.837 | 0.018 | 1.916 | 0.010 | 0.930 | 0.010 |

^1^ Best combination according to ranking criterion 1 ^2^ Best combination according to ranking criterion 2

^3^ Best combination according to ranking criterion 3 ^4^ Best combination according to ranking criterion 4
